# CRISPR/Cas9-Mediated *fech* Knockout Zebrafish: Unraveling the Pathogenesis of Erythropoietic Protoporphyria and Facilitating Drug Screening

**DOI:** 10.3390/ijms251910819

**Published:** 2024-10-08

**Authors:** Hitihami M. S. M. Wijerathna, Kateepe A. S. N. Shanaka, Sarithaa S. Raguvaran, Bulumulle P. M. V. Jayamali, Seok-Hyung Kim, Myoung-Jin Kim, Sumi Jung, Jehee Lee

**Affiliations:** 1 Department of Marine Life Sciences & Center for Genomic Selection in Korean Aquaculture, Jeju National University, Jeju 63243, Republic of Korea; sarathm@ocu.ac.lk (H.M.S.M.W.);; 2 Department of Aquaculture and Seafood Technology, Faculty of Fisheries and Ocean Sciences, Ocean University of Sri Lanka, Colombo 01500, Sri Lanka; 3 Marine Life Research Institute, Jeju National University, Jeju 63333, Republic of Korea; 4 Nakdonggang National Institute of Biological Resources, Sangju-si 37242, Republic of Korea; 5 Marine Molecular Genetics Lab, Jeju National University, 102 Jejudaehakno, Jeju 63243, Republic of Korea

**Keywords:** zebrafish, ferrochelatase, CRISPR/Cas9, erythropoietic protoporphyria, protoporphyrin IX

## Abstract

Erythropoietic protoporphyria (EPP1) results in painful photosensitivity and severe liver damage in humans due to the accumulation of fluorescent protoporphyrin IX (PPIX). While zebrafish (*Danio rerio*) models for porphyria exist, the utility of ferrochelatase (*fech*) knockout zebrafish, which exhibit EPP, for therapeutic screening and biological studies remains unexplored. This study investigated the use of clustered regularly interspaced short palindromic repeats (CRISPR)/Cas9-mediated *fech*-knockout zebrafish larvae as a model of EPP1 for drug screening. CRISPR/Cas9 was employed to generate *fech*-knockout zebrafish larvae exhibiting morphological defects without lethality prior to 9 days post-fertilization (dpf). To assess the suitability of this model for drug screening, ursodeoxycholic acid (UDCA), a common treatment for cholestatic liver disease, was employed. This treatment significantly reduced PPIX fluorescence and enhanced bile-secretion-related gene expression (*abcb11a* and *abcc2*), indicating the release of PPIX. Acridine orange staining and quantitative reverse transcription polymerase chain reaction analysis of the *bax*/*bcl2* ratio revealed apoptosis in *fech*^−/−^ larvae, and this was reduced by UDCA treatment, indicating suppression of the intrinsic apoptosis pathway. Neutral red and Sudan black staining revealed increased macrophage and neutrophil production, potentially in response to PPIX-induced cell damage. UDCA treatment effectively reduced macrophage and neutrophil production, suggesting its potential to alleviate cell damage and liver injury in EPP1. In conclusion, CRISPR/Cas9-mediated *fech*^−/−^ zebrafish larvae represent a promising model for screening drugs against EPP1.

## 1. Introduction

Erythropoietic protoporphyria (OMIM: erythropoietic protoporphyria [EPP1], #177000) is characterized by the accumulation of fluorescent protoporphyrin IX (PPIX) in red blood cells and the liver due to the absence of the ferrochelatase (*fech*) gene, resulting in painful photosensitivity and severe liver damage in humans [1]. PPIX accumulation is predominantly observed in the bone marrow and liver in humans [2]. The primary clinical symptom of patients with EPP1 is photosensitivity arising from circulating or accumulated PPIX in the dermal blood vessels or skin, respectively [3]. In some patients, severe liver damage can occur owing to the toxic effects of accumulated PPIX on liver function and structure, often necessitating liver transplantation [1]. In a previous metabolic analysis on mice, the absence of Fech resulted in the accumulation of bile acids and ceramides in the liver, which are hepatotoxic [4]. Additionally, high levels of phosphatidylcholine, which is crucial for PPIX solubility, were found, [4].

Fech, the terminal enzyme in the heme biosynthesis pathway of cells, inserts ferrous iron into PPIX to produce heme (iron PPIX) [5]. A partial deficiency of Fech leads to the development of EPP1 in humans [6,7]. Moreover, Fech has been identified in the inner mitochondrial membrane of eukaryotes, with its active site facing the mitochondrial matrix, as well as in the cytoplasmic membrane of prokaryotes [5,8]. The protein is 423 amino acids in length and has a molecular weight of 46.95 kDa, and the corresponding gene is 1.296 kbp in length, comprising 11 exons [9]. Additionally, Fech samples extracted from various species exhibit highly similar amino acid sequences, with a common catalytic core of approximately 300 amino acids [8]. Eukaryotic Fech proteins also contain an extra stretch of 30–50 amino acids at the C-terminus and an extension of 30–80 amino acids at the N-terminus [8].

Ursodeoxycholic acid (UDCA), a therapeutic agent used to treat cholestatic hepatopathies and various other hepatic diseases, reduces apoptosis by suppressing mitochondrial pore formation [10]. Additionally, UDCA diminishes bile acid cytotoxicity and enhances renal excretion by regulating the expression of metabolic enzymes and transporter genes [10,11]. Furthermore, UDCA induces the expression of adenosine triphosphate (ATP)-binding cassette subfamily B member 11 (ABCB11 or Bsep) and ATP-binding cassette subfamily C member 2 (ABCC2 or Mrp2), which are primary bile-acid transporter genes, activating the Farnesoid X receptor (FXR), thereby enhancing the secretion of toxic bile acids from hepatocytes [11]. Moreover, a previous study indicated that inhibitors of the bile-acid transporter gene ATP-binding cassette subfamily G member 2 (ABCG2), such as genistein and isoflavone, reduce the fluorescence intensity of PPIX in brain tumor cells, suggesting that activating bile-acid transporter genes may enhance the cellular efflux of PPIX accumulated in hepatic cells, thereby protecting against liver injury [12]. Therefore, in the case of EPP1 with significant liver involvement, UDCA may be used to help manage PPIX accumulation and support the normalization of hepatic function [13]. Furthermore, a case report indicated that administration of UDCA, in conjunction with a discontinuation of iron supplementation, resulted in a complete normalization of hepatic function in EPP1 patients five months after starting the treatment [14]. However, the evidence supporting its beneficial effects in regard to EPP1 remains limited, and there are concerns among experts about the potential adverse effects of this treatment [15]. Further research is needed to clarify the role of UDCA in managing EPP1 and establish its safety and efficacy.

The use of zebrafish (*Danio rerio*) as an animal model for genetic studies was first introduced by Streisinger et al. in the 1980s [16]. Initially, mutagenesis in zebrafish was performed using large-scale N-ethyl-N-nitrosourea (ENU) mutagenesis techniques, followed by thorough phenotypic screening [17]. However, owing to the time-consuming and laborious nature of the ENU technique, with advancements in zebrafish genome mapping, more advanced techniques, such as the use of zinc finger nucleases (ZFNs), transcription activator-like effector nucleases (TALENs), and clustered regularly interspaced short palindromic repeats (CRISPRs) with CRISPR-associated protein 9 (Cas9), have been introduced [18,19,20]. Furthermore, CRISPR/Cas9 has become an efficient method for developing knockout zebrafish models for research [21]. Moreover, the zebrafish model is an attractive tool for studying genetics, development, embryology, cell biology, and drug screening [22]. Zebrafish embryos are distinctive because of their short reproductive cycle, the ease with which they can be maintained and administered drugs, and transparency, the last of which allows the visual inspection of developing cells and organs [22].

Various zebrafish models have been investigated for different types of porphyria disorders, including variegate porphyria (the hepatic form of porphyria) [23], hepatoerythropoietic porphyria (a genetic disorder resulting from a deficiency of the uroporphyrinogen decarboxylase enzyme) [24], and EPP1 [25]. Variegate porphyria occurs due to a deficiency in protoporphyrinogen oxidase (PPOX) [26]. Hepatoerythropoietic porphyria is caused by a mutation in the gene encoding uroporphyrinogen decarboxylase [27]. EPP1 is caused by a deficiency in the fech gene, as described above. In the present study, we discuss the disease EPP1. A previous study described a *fech* mutant zebrafish embryo developed using a less efficient ENU technique known as Dracula (drc) and investigated the liver accumulation of PPIX, light sensitivity, light-dependent hemolysis, and liver disease in the model [25]. However, the mortality of the larvae during their later developmental stages coupled with their sensitivity to light may limit the application of this mutant. Nonetheless, the possibility of using fech-knockout zebrafish as a model for screening therapeutic agents and conducting biological studies has not yet been examined. The present study aimed to explore the potential use of CRISPR/Cas9-mediated fech-knockout zebrafish larvae for screening drugs to alleviate the symptoms of EPP1 and investigate the effectiveness of UDCA as a treatment for managing EPP1 symptoms, particularly in the case of liver involvement.

## 2. Results

### 2.1. Analysis of Fech Expression in Different Larval Stages and Tissues of Zebrafish

**Figure 1:**
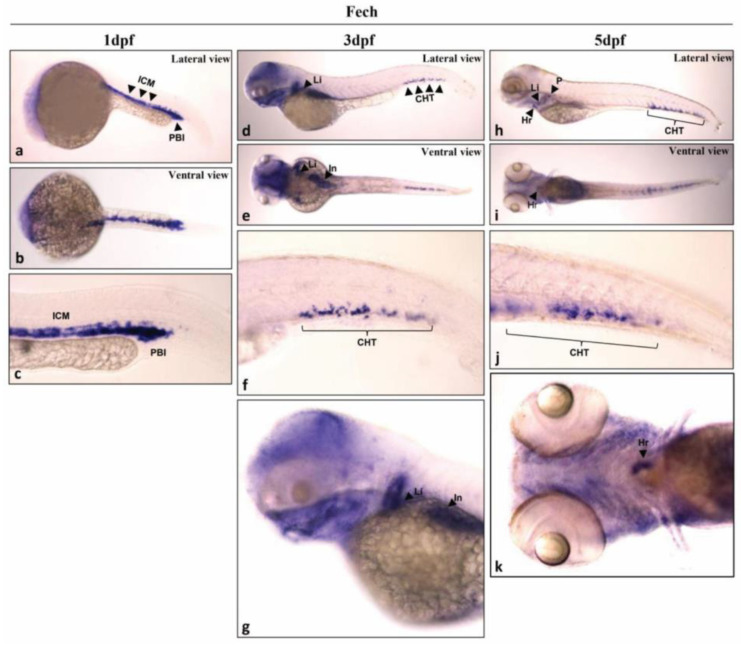
Spatial distribution of *fech* in zebrafish larvae. *fech* expression in larvae 1 day ((**a**) lateral view; (**b**) ventral view; and (**c**) tail), 3 days ((**d**) lateral view; (**e**) ventral view; (**f**) tail; (**g**) head), and 5 days ((**h**) lateral view; (**i**) ventral view; (**j**) tail; (**k**) head) days post-fertilization (dpf). ICM, intermediate cell mass; PBI, posterior blood island; Li, liver; CHT, caudal hematopoietic tissue; Hr, heart; P, pronephros; In, intestine.

To investigate the spatial distribution of *fech* in the zebrafish larvae 1, 3, and 5 days post-fertilization (dpf), a whole-mount in situ hybridization (WISH) analysis was performed. At 1 dpf, *fech* was highly expressed in erythrocytes located in the intermediate cell mass (ICM) and posterior blood island (PBI) (Figure 1a–c). In the 3 dpf larvae, fech was localized to the liver and caudal hematopoietic tissue (CHT) (Figure 1d–g). Similarly, at 5 dpf, *fech* remained localized in the liver and the CHT (Figure 1h–k). In addition, *fech* expression was observed in the heart (Figure 1h,i,k) and pronephros (Figure 1h). These findings provide valuable insights into the dynamic expression pattern of fech during different stages of zebrafish embryo and larvae development.

**Figure 2:**
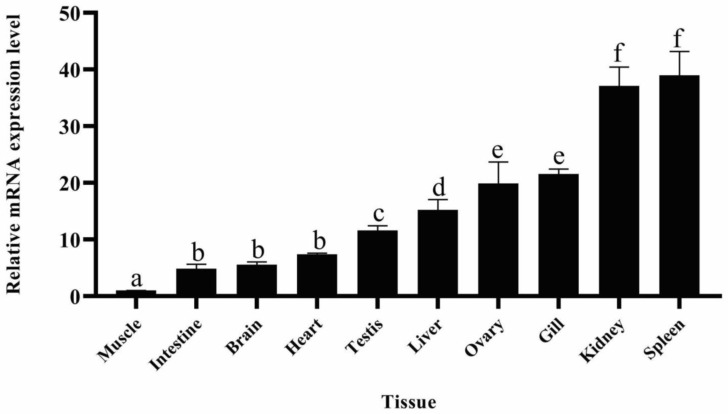
Tissue-specific expression of *fech* in healthy adult zebrafish. The relative mRNA levels of *fech* were assessed in various organs, with muscle tissue used as a reference. The spleen and kidneys exhibited the highest expression, followed by relatively higher levels in the gill, ovary, and liver than in other tissues. Each bar on the graph represents the mean relative mRNA level, with error bars indicating the SD (*n* = 3). Statistical significance was determined using a one-way ANOVA with Tukey’s post hoc test, and different letters indicate statistically significant differences (*p* < 0.05) between tissue types.

The tissue-specific distribution of fech in healthy adult zebrafish (except in blood) was evaluated using quantitative reverse transcription–polymerase chain reaction (RT-qPCR) (Figure 2). The highest fech expression was observed in the spleen and kidneys.

### 2.2. Generation of a Fech-Knockout Zebrafish Model Using CRISPR/Cas9 Gene Editing

**Figure 3:**
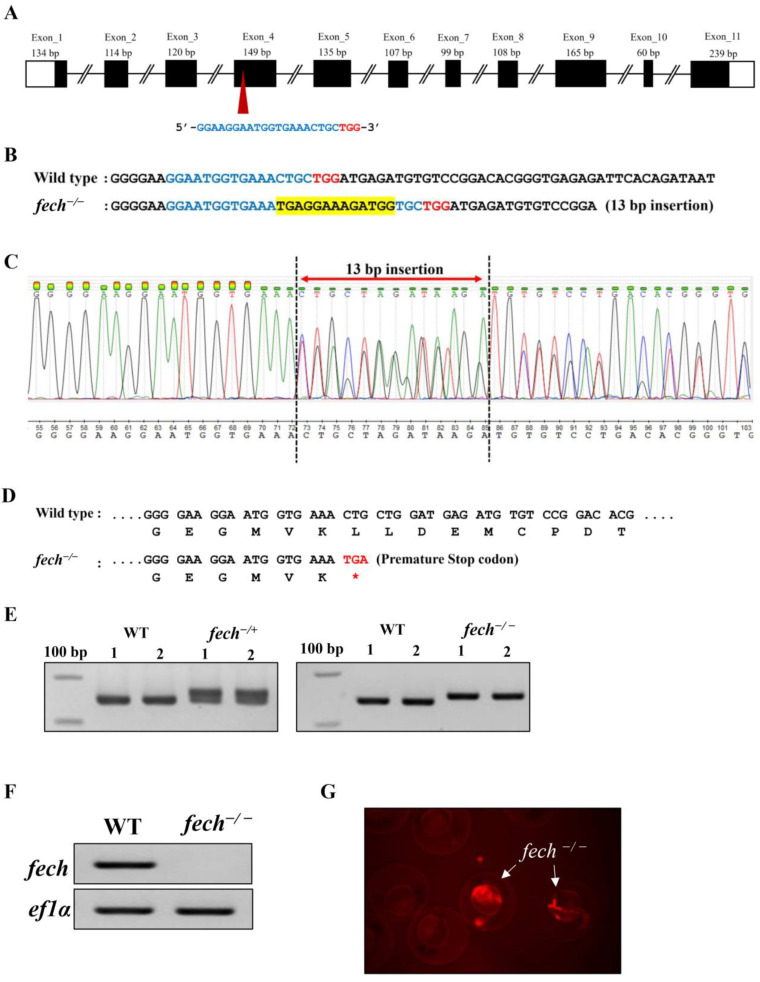
Generation and phenotyping of *fech* ^−/+^ fish and *fech*^−/−^ larvae. (**A**) Schematic representation of the organization of the zebrafish *fech* gene. Untranslated areas and open reading frames are depicted by white and black boxes, respectively. The target location for the sgRNA is denoted by the red arrowhead. The PAM and single-guide RNA (sgRNA) target sequences are indicated by red and blue letters, respectively. (**B**–**D**) Illustration of the insertion of 13 bp nucleotides into the target site and the introduction of a premature stop codon (*) using CRISPR/Cas9 gene editing. The inserted amino acid sequence is highlighted in yellow. (**E**) Genotyping of *fech*^−/+^ and *fech*^−/−^ using PCR and agarose gel electrophoresis. (**F**) Confirmation of the mutation via RT-qPCR using *fech* target-site-specific primers in 7 dpf WT and *fech*^−/−^ larvae. (**G**) Detection of red fluorescence in 24 hpf *fech*^−/−^ larvae during the screening process.

The sgRNA targeting site for the CRISPR/Cas9-mediated knockout of the zebrafish *fech* gene was located within exon 4, corresponding to domain 1 (Figure 3A). The knockout was achieved by introducing a random 13 bp insert into the target site, resulting in a premature stop codon (TGA) (Figure 3B–D). This mutation led to the deletion of approximately half of domain 1 and subsequent domains, encompassing domain 2 and the binding motif of the [2Fe-2S] cluster. Genotyping using short primers revealed a double band in heterozygous *fech*-knockout fish (*fech*^−/+^) and a single upper band in homozygous *fech*-knockout larvae (*fech*^−/−^) (Figure 3E). RT-qPCR analysis of the target site revealed the absence of *fech* transcription in *fech*^−/−^ larvae (Figure 3F).

Notably, *fech*^−/−^ larvae exhibited fluorescence during the late embryonic and whole-larval stages, and this property was utilized as a screening marker for identifying *fech*^−/−^ larvae after breeding *fech*^−/+^ fish (Figure 3G). The red fluorescence was attributed to PPIX accumulation [25]. However, it should be noted that the *fech*^−/−^ larvae perished after 9 dpf. Nonetheless, the current study focused on using *fech*^−/−^ larvae for further investigations.

### 2.3. Phenotypic and Physiological Effects of Fech Deletion in Zebrafish Larvae

Prior to conducting the experiment, we observed phenotypic alterations in the *fech*^−/−^ zebrafish larvae that were bred (Figure S2). In particular, distinguishable deformities in the body were identified in the *fech*^−/−^ larvae 6 dpf onward. Furthermore, reddish, enlarged livers with several red-brown inclusions, probably due to PPIX accumulation, were observed in the larvae. Additionally, the *fech*^−/−^ larvae exhibited yolk sac edema, an inward spinal curvature, and swim bladder abnormalities (uninflated swim bladders) (Figure S2).

When using zebrafish larvae in experiments, it is crucial to observe them under a light microscope. Therefore, analyzing the tolerance of our *fech*^−/−^ larvae to white light emitted by a standard microscope was necessary. To evaluate this, we exposed 1, 3, 5, 7, and 9 dpf wild-type (WT) and *fech*^−/−^ larvae obtained after crossing *fech*^+/−^ males and females to white light from a standard microscope for 20 min. Another set of WT and *fech*^−/−^ larvae remained unexposed, serving as the control group. After exposure, images of the larvae were captured to investigate the acute effects of exposure to light from the microscope on the *fech*^−/−^ larvae (Figure S3). Genotyping was performed after imaging to distinguish between the WT *fech*^+/−^ and *fech*^−/−^ larvae. No additional discernible morphological defects were observed in the *fech*^−/−^ larvae that were exposed compared with those in larvae that were not exposed. This finding confirms that exposing *fech*^−/−^ larvae to microscope light does not induce any additional acute effects on the typical morphological defects observed in *fech*^−/−^ larvae.

Moreover, it is crucial to consider both the acute and chronic effects of light exposure when employing a diseased zebrafish larval model for experimental purposes. Therefore, to investigate the effect of chronic light exposure on *fech*^−/−^ larvae, we exposed seven sets of WT and *fech*^−/−^ larvae to white light from a microscope for 20 min, with each set at a different developmental stage (1, 2, 3, 4, 5, 6, and 7 dpf) for 20 min. Another set of WT and *fech*^−/−^ larvae was exposed on a daily basis from 1 dpf to 7 dpf for 20 min. Larvae in the control group were not exposed. Finally, at 7 dpf, all larvae were examined for morphological defects, and corresponding images were captured (Figure S4). No significant differences outside of the usual morphological changes were observed between the light-exposed and unexposed larvae.

The absence of Fech may affect functional hemoglobin production in erythrocytes, as it is involved in heme synthesis. To confirm whether functional hemoglobin production was suppressed in our *fech*^−/−^ knockout model, we performed *o*-dianisidine staining (Figure S5A). For this, 2–6 dpf WT and *fech*^−/−^ larvae were stained, and brownish-red erythrocytes containing functional hemoglobin were observed only in the WT larvae. However, no staining was observed in the *fech*^−/−^ larvae, indicating that deleting *fech* suppressed heme biosynthesis in erythrocytes.

Weak blood circulation has been reported in *fech*-mutant zebrafish larvae [25]. Correspondingly, in this study, using light microscopy, we observed diminished blood circulation. To confirm whether *fech* deletion indeed suppressed heme production while maintaining erythrocyte production, we performed RT-qPCR to analyze the mRNA levels of GATA binding protein 1 (*gata1*) in 1–6 dpf WT and *fech*^−/−^ zebrafish larvae raised in complete darkness (Figure S5B). As expected, RT-qPCR analysis revealed that *gata1* expression was lower in the *fech*^−/−^ larvae than in the WT larvae. This decrease may be attributed to PPIX accumulation in erythrocytes, which disturbs erythropoiesis. However, the initial gata1 mRNA levels in the 1 dpf WT and *fech*^−/−^ larvae were not significantly different. Starting from 2–3 dpf, a gradual decrease in *gata1* expression was observed, followed by an increase at 4 dpf. As the kidneys become the primary site for erythropoiesis at 4 dpf [28], the sudden upregulation of *gata1* expression could be attributed to the onset of fresh erythropoiesis in the kidneys. However, expression levels remained relatively unchanged in the 5 and 6 dpf larvae, although the levels were consistently lower than those in the WT larvae. Taken together, these results demonstrate that in *fech*^−/−^ larvae, erythrocyte production is continuous; however, the erythrocytes produced are minimal and may be non-functional owing to the absence of heme-containing hemoglobin.

### 2.4. The Dynamics of PPIX Accumulation in fech^−/−^ Larvae at Various Life Stages

**Figure 4:**
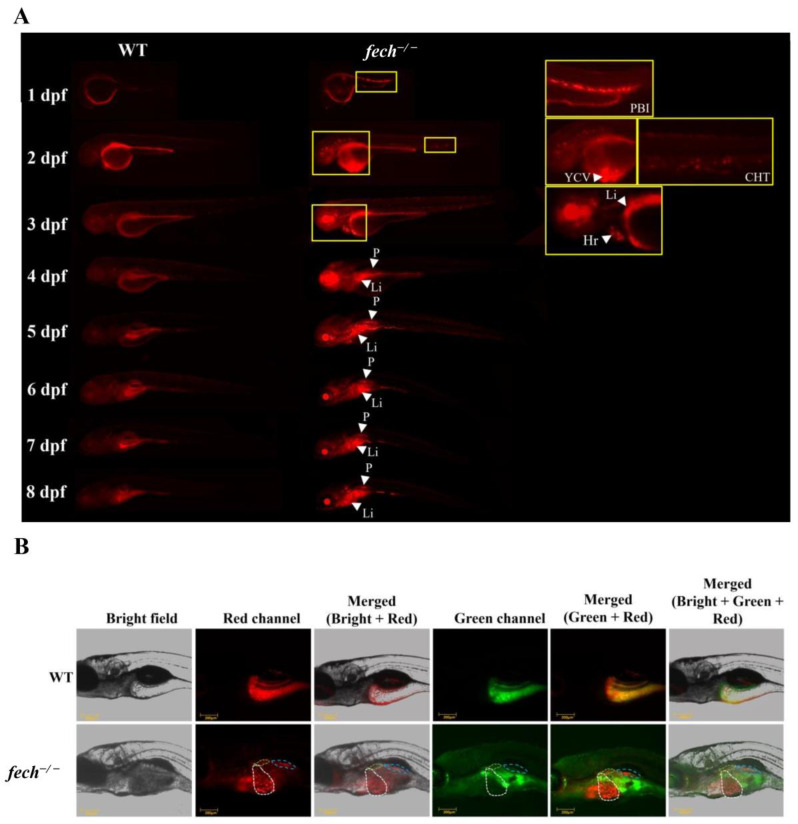
PPIX accumulation in *fech*^−/−^ larvae. (**A**) Fluorescence images of *fech*^−/−^ zebrafish larvae across various developmental stages (PBI, posterior blood island; YCV, yolk circulation valley; CHT, caudal hematopoietic tissue; Li, liver; Hr, heart; P, pronephros). (**B**) PPIX accumulation in 6 dpf zebrafish larvae. Images were taken in a bright field, with the red channel used to detect PPIX fluorescence and the green channel used to detect autofluorescence. Merged images show the localization of PPIX in the liver (white dotted line), pronephros (green dotted line), and intestinal area (blue dotted line).

PPIX exhibits the ability to fluoresce owing to π-electron delocalization in its conjugated double-bond system [29]. Therefore, we examined the distribution of PPIX fluorescence at various developmental stages in *fech*^−/−^ larvae (Figure 4A). As anticipated, red PBI fluorescence was observed in the 1 dpf larvae. In the 2 dpf larvae, PPIX accumulation was predominantly observed in the CHT and the yolk circulation valley (YCV), confirming its accumulation in erythroid tissues. Notably, in the 3 dpf larvae, PPIX accumulation was detected near the liver (Li) and heart (Hr). Subsequently, from 4 dpf onwards, PPIX accumulation in the erythrocytes within the circulation became undetectable, whereas it was observed in the pronephric region (the early kidney) (P) and liver (Li) (Figure 4A,B).

### 2.5. Attenuation of PPIX Accumulation in fech^−/−^ Larvae following UDCA Treatment

**Figure 5:**
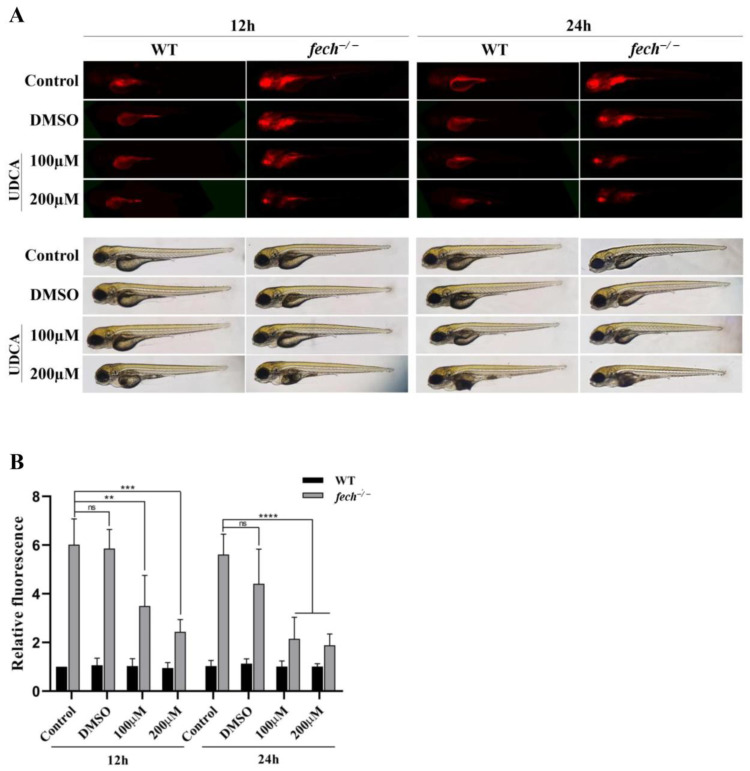
Reduction in PPIX accumulation in *fech*^−/−^ larvae following UDCA treatment. WT and *fech*^−/−^ larvae (3 dpf) were treated with 100 or 200 µM of UDCA, and their PPIX fluorescence intensity was compared with that of untreated WT and *fech*^−/−^ larvae. (**A**) Fluorescence and bright-field images of WT and *fech*^−/−^ larvae after UDCA treatment as well as the controls. (**B**) The relative fluorescence intensities of PPIX in the experimental larvae. The relative fluorescence intensity data are presented as the means ± SD (*n* = 5). The statistical significance between control and treated larvae was analyzed using the Student’s *t*-test (ns, non-significant; **, *p* ≤ 0.01; ***, *p* ≤ 0.001; ****, *p* ≤ 0.0001).

UDCA is used as a therapeutic agent to manage liver damage resulting from PPIX accumulation in EPP1 patients [1]. Therefore, we investigated whether UDCA directly reduced PPIX accumulation in the liver (Figure 5). To achieve this, we administered 100 or 200 µM of UDCA to 3 dpf WT and *fech*^−/−^ larvae for 12 and 24 h, as previous results indicated that PPIX accumulation occurred in zebrafish livers from 3 dpf onward (Figure 5). Another set of WT and *fech*^−/−^ larvae was treated with dimethyl sulfoxide (DMSO), which was the medium used to reconstitute the UDCA powder. The control group did not receive any treatment. Fluorescence images were captured to assess the intensity of PPIX in the larvae. Interestingly, the fluorescence results revealed a lower intensity in the UDCA-treated larvae, suggesting reduced PPIX accumulation in the *fech*^−/−^ larvae compared with that in the untreated *fech*^−/−^ larvae at both time points (Figure 5A,B). However, bright-field images indicated increased yolk sac utilization in both the 200 µM UDCA-treated WT and *fech*^−/−^ larvae, signifying increased metabolism due to UDCA treatment (Figure 5A). Therefore, we opted for the 100 µM UDCA treatment for future experiments.

### 2.6. Suppression of Apoptosis by UDCA Treatment of fech^−/−^ Larvae

**Figure 6:**
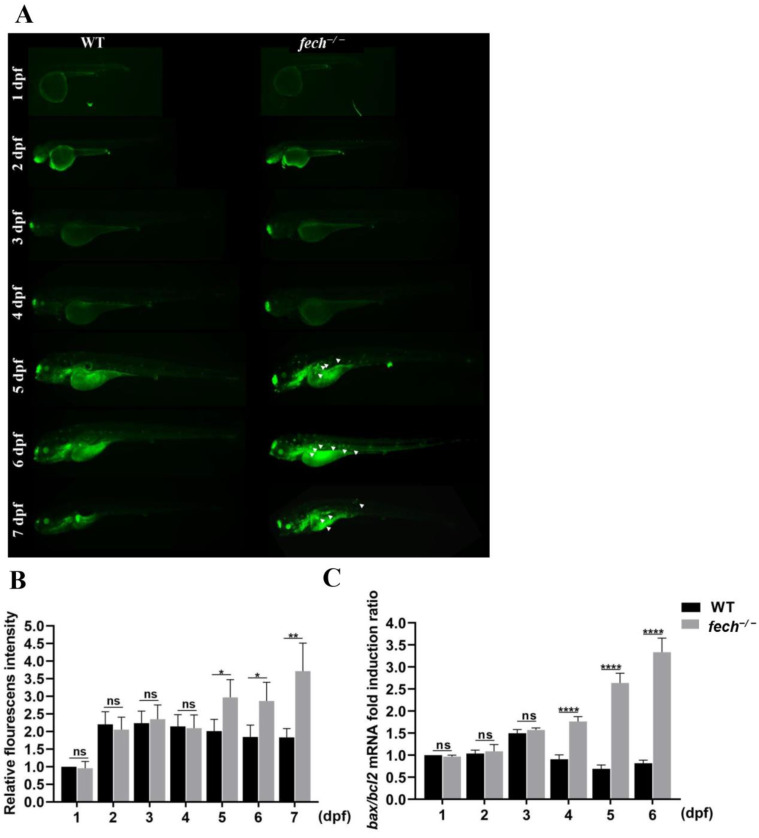
Apoptosis activation in *fech*^−/−^ larvae. (**A**) Acridine orange staining of WT and *fech*^−/−^ larvae at various developmental stages and (**B**) relative fluorescence intensity. (**C**) The *bax/bcl2* mRNA fold induction ratios in WT and *fech*^−/−^ larvae at different developmental stages. The fold induction ratios are presented as the means ± SD (*n* = 3). Statistical significance between *fech*^−/−^ and WT larvae was analyzed using Student’s *t*-test (ns, non-significant; *, *p* ≤ 0.1; **, *p* ≤ 0.01; ****, *p* ≤ 0.0001).

Previous studies have demonstrated that UDCA protects the plasma membrane from cytolysis induced by tensioactive bile acid accumulation during cholestasis [30]. To investigate whether treating *fech*-knockout zebrafish larvae with UDCA suppressed the activation of apoptosis and subsequent cell death, we initially examined the stages at which apoptotic cells could be visualized through acridine orange staining in *fech*^−/−^ larvae. WT and *fech*^−/−^ larvae, at various developmental stages ranging from 1 to 7 dpf, were stained with acridine orange (Figure 6A). The results showed the presence of apoptotic cells in *fech*^−/−^ larvae from 5 dpf onward (Figure 6A,B). Moreover, we analyzed the relative fold induction ratio of BCL2-associated X (*bax*)/B-cell lymphoma 2 (*bcl2*) (intrinsic-apoptosis-pathway-related genes) mRNA. A significant increase in the *bax/bcl2* ratio was observed in *fech*^−/−^ larvae starting at 4 dpf (Figure 6C), indicating the activation of the apoptosis pathway.

**Figure 7:**
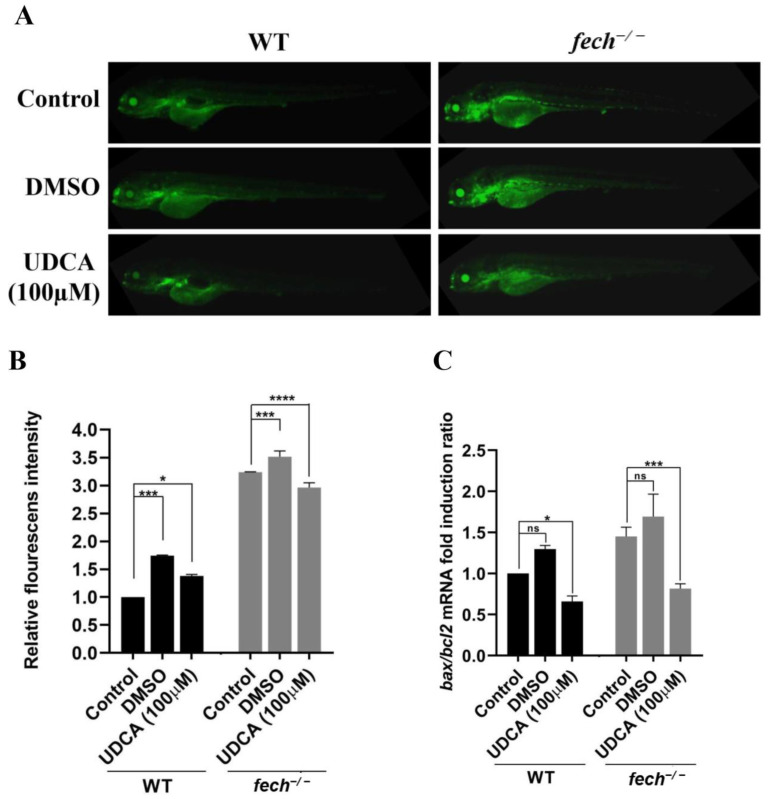
Effect of UDCA treatment on apoptosis in *fech*^−/−^ larvae. (**A**) Acridine orange staining of WT and *fech*^−/−^ larvae after UDCA treatment and (**B**) relative fluorescence intensity. (**C**) The *bax/bcl2* expression ratio after UDCA treatment. The *bax/bcl2* fold induction ratios are presented as the means ± SD (n = 3). Statistical significance between *fech*^−/−^ and WT larvae was analyzed using Student’s *t*-test (ns, non-significant; *, *p* ≤ 0.1; ***, *p* ≤ 0.001; ****, *p* ≤ 0.0001).

Thereafter, 3 dpf *fech*^−/−^ larvae were treated with 100 µM of UDCA, and the *bax/bcl2* expression ratio was analyzed at 4 dpf (1 d post-treatment (dpt)), and acridine orange staining was performed at 5 dpf (2 dpt) to visualize the effect of UDCA on cell apoptosis and the suppression of the apoptosis pathway, respectively (Figure 7). Acridine orange staining showed reduced amounts of green fluorescence in the UDCA-treated *fech*^−/−^ larvae compared with those in the control and DMSO-treated larvae (Figure 7A,B). Similarly, the *bax/bcl2* expression ratio was significantly reduced in UDCA-treated larvae compared with that in control and DMSO-treated larvae (Figure 7C).

Taken together, these results reveal that UDCA may reduce apoptosis activation in *fech*^−/−^ larvae.

### 2.7. Activation of Bile Transportation-Related Genes by UDCA Treatment

**Figure 8:**
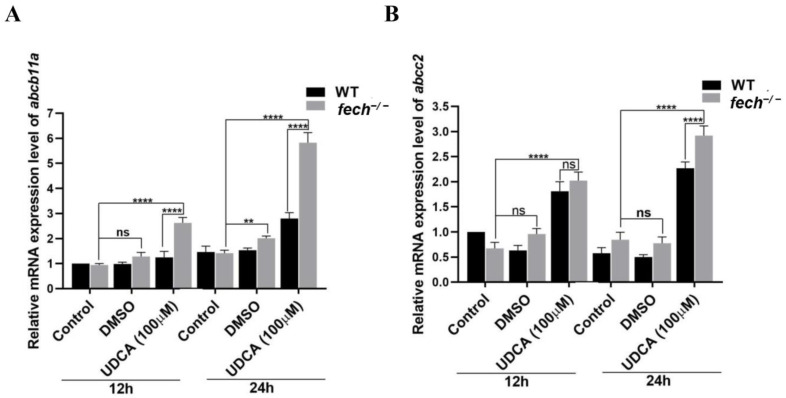
Effect of UDCA treatment on the expression of bile transporters in WT and *fech*^−/−^ larvae (3 dpf) that were treated with 100 µM of UDCA and for which, after 12 and 24 h, qPCR was performed. UDCA treatment induced the expression of (**A**) *abcb11a* and (**B**) *abcc2* to a greater extent in both WT and *fech*^−/−^ larvae compared with that in the control. RT-qPCR results are presented as the means ± SD (*n* = 3). Statistical significance between control and UDCA-treated *fech*^−/−^ larvae was analyzed using the Student’s *t*-test (ns, non-significant; **, *p* ≤ 0.01; ****, *p* ≤ 0.0001).

UDCA induces bile acid transporters, reducing cytotoxicity and improving renal excretion [11]. Based on this, we investigated the effect of UDCA on the primary bile acid transporters *abcb11a* (*bsep*) and *abcc2* (multi-drug-resistance protein 2 (*mrp2*)). For this investigation, 3 dpf WT and *fech*^−/−^ larvae were treated with 100 µM of UDCA, whereas another group was treated with DMSO, and the control group remained untreated for 24 h. RT-qPCR was then performed to analyze the *abcb11a* and *abcc2* mRNA levels (Figure 8). UDCA treatment induced the expression of both transporters in the WT and *fech*^−/−^ larvae. Moreover, the expression of *abcb11a* and *abcc2* in UDCA-treated *fech*^−/−^ larvae was higher than that in the WT larvae. These results suggest that UDCA treatment enhances the expression of bile acid transporters.

### 2.8. Temporal Changes in Neutrophil Production and Attenuation of Neutrophil Accumulation via UDCA Treatment in fech^−/−^ Larvae

**Figure 9:**
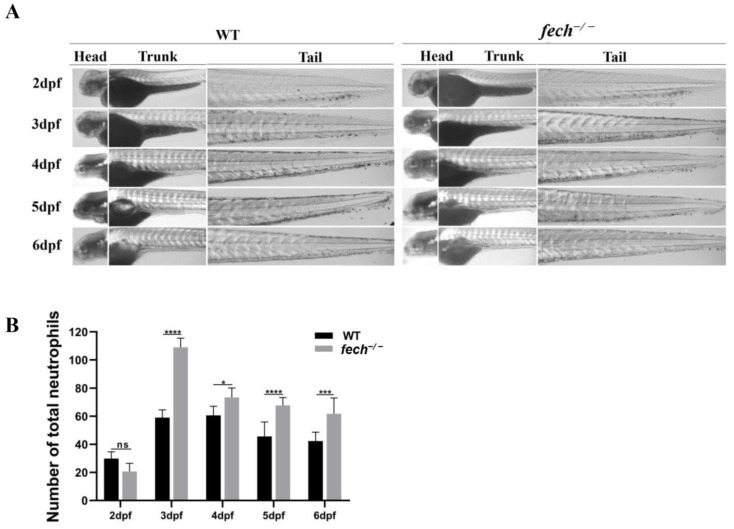
Temporal neutrophil production in *fech*^−/−^ larvae. (**A**) Changes in neutrophil production in the head, trunk, and tail of WT and *fech*^−/−^ zebrafish larvae at different developmental stages. (**B**) The total neutrophil counts in WT and *fech*^−/−^ larvae at various developmental stages. The highest neutrophil count in *fech*^−/−^ larvae compared to the WT was observed at 3 dpf. The total neutrophil counts are presented as the means ± SD (*n* = 5). Statistical significance between WT and *fech*^−/−^ larvae was analyzed using Student’s *t*-test (ns, non-significant; *, *p* ≤ 0.1; ***, *p* ≤ 0.001; ****, *p* ≤ 0.0001).

Liver injury triggers an inflammatory response that leads to the recruitment and activation of neutrophils [31]. Therefore, we first investigated the pattern of neutrophil production in *fech*^−/−^ zebrafish larvae at different developmental stages (Figure 9A,B). Sudan black staining was employed to visualize neutrophils at 2–6 dpf in WT and *fech*^−/−^ zebrafish larvae (Figure 9A), and the total number of neutrophils observed in whole larvae was quantified (Figure 9B). No significant differences in neutrophil numbers were observed between the WT and *fech*^−/−^ 2 dpf larvae. However, in the 3–6 dpf larvae, a higher number of neutrophils was observed in the *fech*^−/−^ larvae than in the WT larvae, and the highest increase in neutrophils was observed in the 3 dpf *fech*^−/−^ larvae (Figure 9A,B).

**Figure 10:**
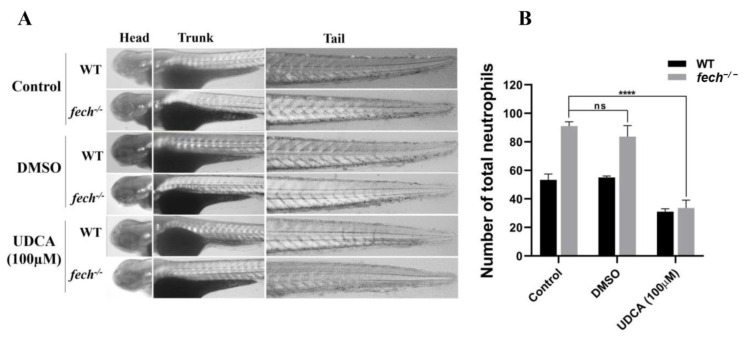
Amelioration of neutrophil accumulation by UDCA treatment in *fech*^−/−^ larvae. (**A**) Images of Sudan black-stained neutrophils in WT and *fech*^−/−^ larvae. (**B**) The total neutrophil counts in WT and *fech*^−/−^ larvae. WT and *fech*^−/−^ larvae (2 dpf) were treated with 100 µM of UDCA, and another group was treated with DMSO for 24 h. The total neutrophil counts are presented as the means ± SD *(n* = 5). Statistical significance between control, DMSO-treated, and UDCA-treated *fech*^−/−^ larvae was analyzed using Student’s *t*-test (ns, non-significant; ****, *p* ≤ 0.0001).

Given the initial increment in neutrophil production observed in the 3 dpf *fech*^−/−^ larvae compared with that in the WT, we treated 2 dpf WT and *fech*^−/−^ larvae with 100 µM of UDCA and another group with DMSO, and the control group remained untreated for 24 h; subsequently, Sudan black staining was performed to visualize and quantify the neutrophils (Figure 10A,B). Notably, the UDCA-treated *fech*^−/−^ larvae exhibited significantly lower neutrophil counts than the control and DMSO-treated *fech*^−/−^ larvae (Figure 10B).

### 2.9. Temporal Changes in Macrophage Production and Attenuation of Macrophage Accumulation by UDCA Treatment in fech^−/−^ Larvae

**Figure 11:**
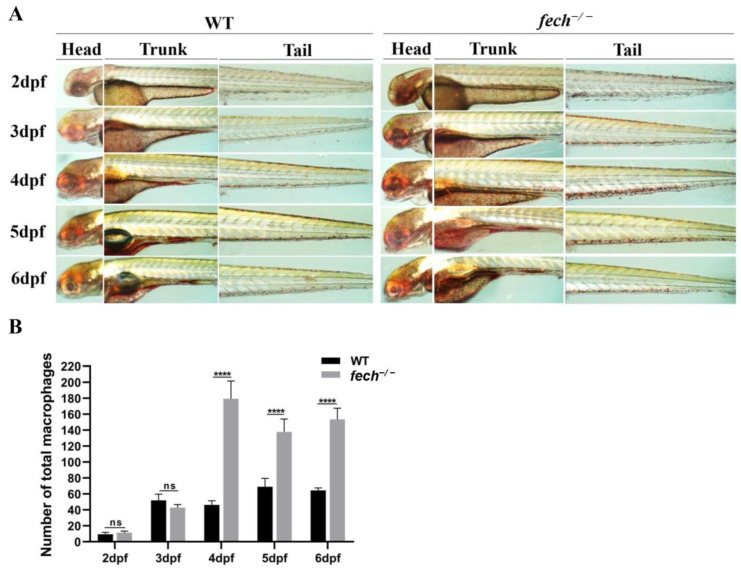
Changes in temporal macrophage production in *fech*^−/−^ larvae. (**A**) Alterations in macrophage production in the heads, trunks, and tails of WT and *fech*^−/−^ zebrafish larvae during different developmental stages. (**B**) The total number of macrophages counted in WT and *fech*^−/−^ larvae at various developmental stages. The highest macrophage counts for *fech*^−/−^ larvae, compared with those for the WT, were observed at 4 dpf. Total macrophage counts are presented as the means ± SD (*n* = 5). Statistical significance between WT and *fech*^−/−^ larvae was analyzed using Student’s *t*-test (ns, non-significant; ****, *p* ≤ 0.0001).

In *fech*^−/−^ larvae, PPIX accumulation in the liver may trigger a cascade of inflammatory responses that subsequently lead to macrophage activation. Therefore, we investigated changes in macrophage production in the *fech*^−/−^ larvae during their developmental stages. To achieve this, we performed neutral red staining to visualize the macrophages in 2–6 dpf WT and *fech*^−/−^ larvae, and the total number of macrophages at each developmental stage was quantified (Figure 11A,B). No significant differences in macrophage numbers were observed between the WT and *fech*^−/−^ larvae until 3 dpf. However, from 4 dpf onward, the *fech*^−/−^ larvae showed higher macrophage numbers than the WT larvae. The highest number of macrophages was observed in the 4 dpf *fech*^−/−^ larvae.

**Figure 12:**
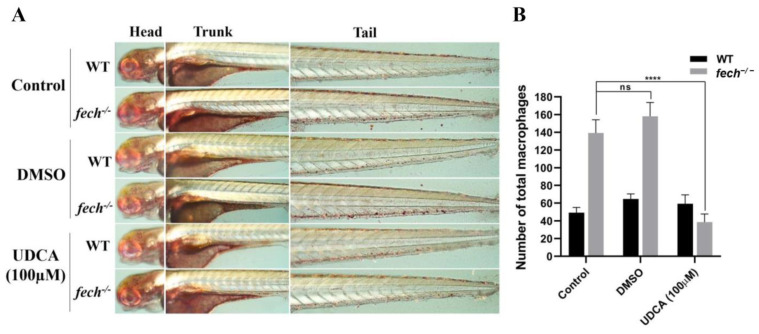
Attenuation of macrophage accumulation by UDCA treatment in *fech*^−/−^ larvae. (**A**) Images of neutral, red-stained macrophages in WT and *fech*^−/−^ larvae. (**B**) The total macrophage counts in WT and *fech*^−/−^ larvae. WT and *fech*^−/−^ larvae (3 dpf) were treated with 100 µM of UDCA, and another group was treated with DMSO for 24 h; the control group remained untreated. The total macrophage counts are presented as the means ± SD (*n* = 5). Statistical significance between the control, DMSO-treated, and UDCA-treated *fech*^−/−^ larvae was analyzed using Student’s *t*-test (ns, non-significant; ****, *p* ≤ 0.0001).

To investigate the effect of UDCA on macrophage production, as a therapeutic strategy, we treated 3 dpf WT and *fech*^−/−^ larvae with 100 µM og UDCA, given the initial increment in macrophage production observed in 4 dpf *fech*^−/−^ larvae. Another group was treated with DMSO, and the control group remained untreated for 24 h. Thereafter, neutral red staining, imaging, and counting of macrophages were performed. UDCA treatment reduced macrophage production compared with that in the control and DMSO-treated *fech*^−/−^ larvae (Figure 12A,B).

## 3. Discussion

The present study was primarily conducted to investigate the pathogenesis of EPP1 and develop a suitable animal model for screening drugs for treating EPP1 in zebrafish using CRISPR/Cas9-mediated gene editing. Because zebrafish serve as an attractive animal model for conducting in vivo experiments across various fields, such as genetics, development, embryology, cell biology, and drug screening, owing to their close genetic resemblance with respect to humans [16], a multiple sequence analysis (Figure S1) and pairwise sequence comparison (Table S2) were conducted, revealing structural similarity between zebrafish and human Fech.

As Fech is an essential component of hemoglobin for O_2_ transportation in the circulation, exploring fech expression patterns in zebrafish embryos offers valuable insights into the temporal and spatial dynamics of hematopoiesis and organogenesis. Previous studies utilizing WISH have provided a foundational understanding of *fech* expression patterns in early-stage zebrafish embryos [25,32]. At the 9–10-somite stage (ss), *fech* expression in the lateral plate mesoderm suggests its early involvement in the development of the pronephric duct and blood vessels [25,32]. Subsequently, *fech* expression is concentrated in the erythrocytes by 24 hpf, underscoring its role in erythropoiesis and heme biosynthesis [25,33]. Our results expand on the findings of these studies, revealing additional sites where *fech* expression can be detected. The expression of *fech* was particularly prominent in the ICM and PBI at 1 dpf. These observations align with those made in previous research, highlighting the significance of these sites in hematopoietic stem cell (HSC) production and blood cell differentiation [34]. Subsequently, the HSCs migrate to the CHT and other locations within the larvae [34,35]. Further investigations at 3 and 5 dpf revealed the dynamic localization of *fech* in the liver and CHT, confirming its role in hematopoiesis and organ development. Additionally, the expression of *fech* in the heart and pronephros in 5 dpf larvae provides valuable insights into the dynamic expression pattern of *fech* during different developmental stages in zebrafish embryos and larvae.

Heme can be produced by all cells, and the regulation of the heme biosynthetic pathway varies depending on the tissue and cell type [36]. As an essential enzyme in heme biosynthesis, Fech plays a vital role in this process. Furthermore, the enzyme heme oxygenase plays a crucial role in maintaining a precise balance between heme production and catabolism for the regulation of cellular heme levels [37]. Therefore, the expression of *fech* may vary between different cell types and tissues. The highest expression of *fech* was clearly observed in erythrocytes [25]. Consequently, a tissue distribution analysis of *fech*, excluding in the blood, in healthy adult zebrafish revealed that the expression of this gene is highest in the spleen and kidneys. The spleen is recognized as a hematopoietic tissue [38], and it has been suggested that the zebrafish spleen could serve as a storage and destruction site for erythrocytes because the absence of Fech in erythrocytes renders them non-functional [39]. The elevated expression of *fech* in the gills and ovaries suggests the occurrence of local heme synthesis to support their metabolic demands. Gills, which are critical for oxygen extraction [40], probably require heme for oxygen transport and as a cofactor for energy metabolism [36,38]. Similarly, ovaries utilize heme extensively for cell proliferation and energy metabolism during oogenesis [41,42]. Hence, heightened *fech* expression in these tissues may be essential for meeting their demand for heme production. Previous studies on mammal (rat) *fech* activity also reported higher expression in the marrow, liver, spleen, and red blood cells [43].

However, it is worth noting that *fech* deficiency does not cause liver disease in most patients with EPP1; rather, the disease occurs only in a small minority, with serious liver disease occurring only in a small percentage of cases over their entire life spans, with an incidence of approximately 2–3% [44], even though *fech* mRNA levels in the zebrafish adult liver are lower than those in the spleen, kidneys, gills, and ovary. The specific reason for this difference has not been extensively investigated. The liver is a vital organ responsible for various critical processes, including metabolism, digestion, detoxification, immunity, and vitamin storage [45]. Therefore, the relatively low *fech* expression observed in this organ can be explained by the fact that heme biosynthesis may not be the primary role of the liver. However, in the whole organ, the liver may express comparatively higher levels of *fech*, as observed in the WISH analysis performed on larvae.

The loss of functional Fech leads to significant phenotypic alterations and physiological disturbances. The most pronounced alteration arises from the accumulation of fluorescent PPIX in the bloodstream and various tissues, resulting in severe photosensitivity and liver damage characteristically associated with EPP1 [44]. Therefore, in this study, we induced EPP1 in zebrafish larvae by knocking out the *fech* gene using the CRISPR/Cas9 gene-editing tool. Subsequently, this EPP1 disease model (*fech*^−/−^ larvae) was used as an animal model for drug screening to alleviate EPP symptoms in zebrafish larvae.

Morphological analysis showed a reddish and enlarged liver due to PPIX accumulation. Similar results were observed by Childs et al. in Fech-encoded Dracula-knockout zebrafish larvae [25]. Moreover, *fech*^−/−^ larvae displayed yolk sac swelling, inward spinal curvature, and abnormalities in the swim bladder, with swim bladders remaining uninflated (Figure S2).

Severe pain upon exposure to the sun, owing to PPIX accumulation, is a common symptom of EPP1 [1,46]. However, only some patients experience sensitivity to artificial light [46], and symptom severity in patients can vary [1]. Childs et al. reported that *fech*-knockout zebrafish larvae were sensitive to standard microscope-derived white light illumination during prolonged exposure (15–20 min) [25]. When conducting experiments with zebrafish larvae, it is essential to observe them using a light microscope. Therefore, the tolerance of our *fech*^−/−^ larvae to acute and chronic exposure to the white light from a standard microscope was examined.

These investigations revealed that our *fech*^−/−^ larvae did not exhibit any significant morphological defects in response to acute or chronic exposure to white light from a standard microscope. Thus, apart from the already existing abnormalities, our *fech*^−/−^ larvae appeared to be resistant to the effects of white light commonly emitted by a standard microscope. However, further studies are required to investigate the reasons underlying these observations.

Hemoglobin is an oxygen-binding protein in erythrocytes and serves as an oxygen-transporting molecule. The two main components of hemoglobin are globin and heme [47]. Therefore, the effects of *fech* deletion on erythropoiesis were investigated using o-dianisidine staining to detect heme production and RT-qPCR analysis to detect *gata1* mRNA expression. Gata1 is critical for the growth and division of immature red blood cells and serves as an erythrocyte marker [48]. Interestingly, our results reveal that *fech* deletion suppresses heme biosynthesis in erythrocytes and *gata1* expression still occurs in *fech*^−/−^ larvae, albeit to a lower degree than in WT larvae. In summary, our findings suggest that while erythrocyte production continues in *fech*^−/−^ larvae, the resulting erythrocytes are scarce and ineffective due to the lack of heme-containing hemoglobin.

PPIX, a substrate for Fech, predominantly accumulates in erythroid tissue in patients with EPP1 and binds to albumin in the plasma or to low- or high-density lipoproteins [49]. Although a limited amount of PPIX is ubiquitously present in all living cells as a heme precursor, its levels are tightly regulated because of its toxic effects, and heme biosynthesis is predominantly active only in tissues with high heme utilization [50]. The accumulation of PPIX in human porphyria can cause various complications [50]. PPIX exhibits fluorescence emission properties [29]. In *fech*^−/−^ zebrafish larvae, we observed the distribution of fluorescence in the PBI at 1 dpf and subsequently in the CHT and YCV, which are erythroid tissues, at 2 dpf. At 3 dpf, substantial PPIX accumulation was observed in the liver and heart, which suggests that the liver breaks down non-functional erythrocytes, leading to PPIX accumulation in the liver, given its role in heme biosynthesis and the absence of Fech [4,45]. From 4 dpf onward, no erythrocytes with accumulated PPIX were observed within the circulation; however, they were detectable in the pronephric region, which is the primary hematopoietic tissue in zebrafish and becomes functional after 4 dpf, as well as in the liver and intestinal area. Previous research has also suggested that PPIX accumulates in the pronephric region of 4 dpf *fech*-deficient zebrafish larvae [25]. However, the intestinal localization of PPIX is yet to be elucidated. The absence of detectable PPIX in the heart and circulation implies reduced erythrocyte levels in the circulatory system, whereas its presence in the main erythropoietic tissue (pronephros) and liver aligns with the results of our *gata1* expression analysis (Figure S5B).

UDCA is primarily used to treat cholestatic liver diseases [11]. Its mechanism of action involves protecting damaged cholangiocytes from bile-acid toxicity, stimulating the impaired biliary secretion, promoting the detoxification of hydrophobic bile acids, and inhibiting hepatocyte apoptosis [30]. Ardalan et al. described the use of UDCA as a therapeutic agent for managing liver damage resulting from PPIX accumulation in patients with EPP1 [1]. However, the absence of heme would directly impact the function of cytochrome P450 enzymes, which are involved in the metabolism of xenobiotics and drugs. Despite this, UDCA does not primarily rely on cytochrome P450 enzymes for its metabolism. UDCA is mainly metabolized via conjugation with amino acids, such as glycine and taurine, in the liver [51]. Therefore, we investigated whether UDCA had a direct effect on reducing PPIX accumulation in the livers of our *fech*^−/−^ zebrafish models. Our results showed that UDCA could indeed reduce PPIX accumulation in *fech*^−/−^ zebrafish larvae. Furthermore, a previous study revealed the hepatoprotection ability of UDCA in a transgenic zebrafish model, *Tg* (*pck1:Casper3GR*) [52]. However, the use of high concentrations of UDCA (200 µM) resulted in rapid utilization of the yolk sac. Therefore, 100 µM of UDCA was used for further experiments. Taken together, our results reveal that *fech*^−/−^ larvae can be utilized to observe the attenuation of PPIX accumulation following UDCA treatment.

In addition to protecting plasma membranes from cytolysis induced by bile-acid accumulation [30], UDCA inhibits apoptosis by preventing mitochondrial pore formation, death receptor recruitment to the membrane, and endoplasmic reticulum stress [11]. Acridine orange staining revealed the presence of apoptotic cells in *fech*^−/−^ larvae from 5 dpf onward. However, our PPIX accumulation data indicated that PPIX accumulation in the liver commenced 3 dpf onward. Moreover, a significant increase in the *bax*/*bcl2* (intrinsic-apoptosis-pathway-related genes) ratio in *fech*^−/−^ larvae was observed from 4 dpf. Previous studies have shown that the accumulation of substantial quantities of PPIX in the liver can induce toxic effects, leading to cholestatic liver injury [50]. Therefore, although PPIX accumulation in the liver begins at 3 dpf, the maximum accumulation and toxic effects may only occur after 5 dpf. Considering that the liver is a vital organ responsible for various biological processes within the body [45,53,54], increased PPIX accumulation over time may impair liver function, leading to disruptions in other physiological processes in the body and affecting other organs. Previous studies on humans have indicated that PPIX accumulation can cause biliary stone development, hepatobiliary damage, and liver failure [50]. The cumulative effects of these disruptions may result in an increased number of apoptotic cells, as visualized using acridine orange staining.

Following this, we investigated the effects of UDCA on apoptotic activation and cell death in *fech*^−/−^ larvae. Acridine orange staining revealed that UDCA reduced cell death and activation of the apoptosis pathway (i.e., reduced the *bax*/*bcl2* ratio) in *fech*^−/−^ larvae. Previous studies have also indicated that UDCA activates the anti-apoptotic pathway by binding to the epidermal growth factor receptor (*Egfr*) and subsequently activating phosphoinositide 3 kinase (*Pi3k*), mitogen-activated protein kinase (*Mapk*), extracellular signal-regulated protein kinase (*Erk1/2*), transcription factors, and serum response factor (*Srf*). This activation ultimately leads to the upregulation of anti-apoptotic proteins (Bcl2 and Bcl-XL) and the suppression of pro-apoptotic proteins (Bax) [55,56].

As described in previous studies, UDCA induces the expression of bile acid transporters, thereby reducing cytotoxicity and improving renal excretion [11]. Based on this hypothesis, we investigated the effect of UDCA on the primary bile acid transporters *abcb11a* (*bsep*) and *abcc2* (*mrp2*). The results revealed that UDCA treatment induces the expression of both transporters in both WT and *fech*^−/−^ larvae. Moreover, the expression of *abcb11a* and *abcc2* in UDCA-treated *fech*^−/−^ larvae was higher than that in the WT larvae. Furthermore, UDCA induces the expression of bile acid transport genes in primary human hepatocytes [57].

Previous studies have highlighted the fact that bile duct obstruction due to PPIX accumulation is a major factor in liver failure associated with EPP1 [58,59]. Given its highly hydrophobic nature, PPIX accumulates to a notable extent in the liver [60]. Under normal conditions, the liver eliminates PPIX via the biliary system [61]. Therefore, elevated PPIX production may block the bile duct, leading to liver injury that can progress to fibrosis, cirrhosis, and liver failure. UDCA enhances bile transport by inducing the expression of genes involved in this process, thereby facilitating the clearance of bile and accumulated PPIX from the liver. This action may mitigate the toxic effects that trigger hepatocyte apoptosis, thereby preventing liver injury. However, the higher expression levels in UDCA-treated *fech*^−/−^ larvae than those in WT larvae may be attributed to the increased activation of *abcb11a* and *abcc2*, facilitating the removal of high amounts of accumulated bile and PPIX from the liver. Additionally, a previous study revealed that inhibiting *Abcg2*, another gene in the ABC transporter family, increased PPIX fluorescence in U87MG cells (a specific glioblastoma cell line derived from human malignant glioblastoma multiforme, a type of brain tumor) [12]. This may provide insights into the mechanisms through which UDCA treatment reduces PPIX accumulation in *fech*^−/−^ larvae.

Following liver injury, an inflammatory response is triggered, resulting in the recruitment and activation of neutrophils [31]. In EPP1, PPIX accumulation can induce hepatobiliary damage and liver injury [60]. The release of various inflammatory mediators from damaged liver tissue induces an immune response that results in neutrophil production. In mammals, liver injury can lead to the release of danger-associated molecular patterns (DAMPs) and cytokines that stimulate bone marrow to increase neutrophil production [62].

Therefore, we first investigated the patterns of neutrophil production in *fech*^−/−^ zebrafish larvae at various developmental stages. Sudan black staining indicated that from 3 to 6 dpf, a greater number of neutrophils was observed in *fech*^−/−^ larvae than in WT larvae. The peak increase in neutrophil numbers at 3 dpf may be attributed to the initial signal released by the liver, thereby triggering neutrophil production, as liver development initiates at 3 dpf.

While Sudan black staining revealed increased neutrophil production at 3 dpf, the results of our expression analysis of apoptosis genes (*bax/bcl2* ratio) showed that the apoptosis signaling pathway was activated only after 4 dpf. This observation is consistent with the fact that neutrophil production in zebrafish typically begins prior to the activation of apoptotic genes as neutrophils are rapidly mobilized to affected areas in response to various stresses and infections [63,64]. Therefore, the initiation of PPIX accumulation in the liver may induce cellular stress, potentially inducing a rapid stimulation of neutrophil production, as neutrophils serve as the first responders of the immune system, regulating cellular processes and eliminating damaged or excessive cells [63]. Understanding the dynamics of neutrophil production and activation in the context of liver injury in *fech*^−/−^ larvae (EPP larvae) is crucial to elucidating the pathophysiology of EPP1 and developing potential therapeutic strategies for mitigating the inflammatory response and subsequent liver damage.

Subsequently, we investigated whether UDCA treatment could reduce neutrophil production in *fech*^−/−^ larvae. Remarkably, UDCA reduced neutrophil production in *fech*^−/−^ larvae compared with that in the control and DMSO-treated *fech*^−/−^ larvae.

The mechanism by which UDCA treatment reduces neutrophil accumulation in *fech*^−/−^ zebrafish larvae (EPP larvae) can be attributed to its multifaceted effects on liver function and the inflammatory response. UDCA exhibits anti-inflammatory properties and has been demonstrated to modulate immune responses in various liver pathologies [11,31].

UDCA suppresses the accumulation of bile acids and toxic intermediates [11]. By improving bile transportation and facilitating the removal of bile and accumulated PPIX from the liver, UDCA can alleviate liver stress and inhibit the release of inflammatory signals that stimulate neutrophil production [31,62]. Moreover, UDCA enhances the expression of bile acid transporters to enhance the efficient elimination of bile and PPIX from the liver, thereby suppressing the signals that trigger the inflammatory response [58,59]. Furthermore, the ability of UDCA to stabilize cell membranes and inhibit apoptosis contributes to a reduction in neutrophil accumulation [11]. Therefore, through its combined effects on bile acid transport, liver protection, and anti-apoptotic mechanisms, UDCA effectively mitigates neutrophil accumulation and dampens the inflammatory response associated with liver injury in *fech*^−/−^ zebrafish larvae.

Macrophages are key players in the immune system that are activated in response to tissue damage and infection [65]. They are also important for phagocytosis, antigen presentation, and the secretion of various inflammatory and anti-inflammatory cytokines [66]. In *fech*^−/−^ larvae, PPIX accumulation in the liver triggers a cascade of inflammatory responses that subsequently lead to the activation of macrophages. Therefore, we investigated changes in macrophage production during the various developmental stages of *fech*^−/−^ larvae. Neutral red staining revealed elevated macrophage numbers in *fech*^−/−^ larvae compared with those in the WT larvae from 4 dpf onward. In contrast, neutrophil production was triggered at 3 dpf. Macrophages play a more complex and versatile role in the immune response and are involved in various functions, including phagocytosis, antigen presentation, and cytokine secretion [67]. The delayed increase in the production of macrophages relative to that of neutrophils observed after 4 dpf in the *fech*^−/−^ larvae may indicate a more coordinated and sustained response to accumulating damage and inflammatory signals. In addition, macrophages typically play a key role in resolving inflammation and participate in tissue repair processes following the initial immune response [66]. Understanding the relationship between PPIX accumulation and macrophage production in *fech*^−/−^ larvae is essential for elucidating the pathophysiology of EPP1 and for developing potential therapeutic strategies aimed at controlling the inflammatory response and minimizing liver damage.

Furthermore, after treating *fech*^−/−^ larvae with UDCA, neutral red staining of 3 dpf larvae revealed reduced macrophage production in the UDCA-treated *fech*^−/−^ larvae compared with that in the control *fech*^−/−^ larvae. The reduced macrophage production in the *fech*^−/−^ larvae following UDCA treatment can be attributed to several factors. UDCA has been reported to possess anti-inflammatory properties and modulate immune responses by regulating cytokine production and immune cell activation [68,69]. Previous studies have indicated that UDCA suppresses the activation of macrophages and other immune cells, leading to a decrease in pro-inflammatory responses [11,70]. Furthermore, UDCA has been demonstrated to inhibit the production of various inflammatory mediators, including tumor necrosis factor alpha (TNF-α) and interleukins (such as IL-2 and IL-4), which are known to play a role in the recruitment and activation of macrophages [11].

UDCA exerts protective effects on the liver, which can indirectly reduce macrophage production [11]. By alleviating liver injury and reducing hepatocyte damage, UDCA may mitigate the release of DAMPs and other signaling molecules that typically stimulate macrophage activation. Furthermore, the regulation of bile acid metabolism by UDCA may restore the balance between inflammatory and anti-inflammatory signals within the liver microenvironment, thereby decreasing the infiltration and activation of macrophages. Overall, the diverse anti-inflammatory and hepatoprotective effects of UDCA contributed to the attenuation of macrophage production in the *fech*^−/−^ larvae, highlighting its potential as a therapeutic agent for alleviating the inflammatory response associated with EPP1-related liver injury.

Collectively, these results indicate that the deletion of Fech may not negatively impact hematopoiesis, leading to the production of neutrophils and macrophages. However, the impact of *fech* deletion on liver injury appears to induce an increased production of both neutrophils and macrophages in *fech*^−/−^ larvae. However, although *fech* deletion may not affect hematopoiesis, it affects the function of erythrocytes. This finding underscores the potential utility of *fech*^−/−^ larvae as an animal model for studying hematopoiesis and the immune system dynamics in patients with EPP1, particularly in therapeutic studies and for drug screening to alleviate EPP1 symptoms.

Our findings underscore the potential of the CRISPR/Cas9 fech-knockout zebrafish model in advancing drug screening and genetic disease research, particularly for EPP1. This model effectively mimics critical features of EPP1, such as PPIX accumulation and liver damage, making it a valuable tool for testing new treatments. For instance, the observed effectiveness of UDCA in reducing PPIX levels demonstrates the utility of this model in evaluating drugs aimed at treating EPP1 [11,30,52]. Moreover, by reflecting how genetic alterations impact disease, this model supports the development of personalized treatments tailored to individual patient needs [57,68]. The ability to employ this zebrafish model for high-throughput screening should accelerate the discovery of effective therapies. Overall, it represents a promising approach for enhancing treatment options and advancing personalized medicine for EPP1 and similar genetic disorders.

Furthermore, we explored the impact of Fech deletion on key immunological and hematopoietic processes and assessed the feasibility of using our *fech*^−/−^ zebrafish model for drug screening. This research provides a foundation for future studies aimed at further elucidating the pathogenesis of EPP1 and developing novel therapeutic strategies for EPP1 and related conditions.

## 4. Materials and Methods

### 4.1. Zebrafish Maintenance

Zebrafish were maintained as previously described [71]. Wild-type (WT, AB) strains were used for all experiments. Adult and juvenile zebrafish were raised in a water-recirculating system at a constant temperature of 28 ± 0.5 °C under a 14:10 h light–dark cycle. Embryos were maintained in embryo medium in an incubator at 28 °C until the larvae hatched. All experimental procedures were performed in accordance with the standards established by the Animal Experiment Ethics Committee of Jeju National University (approval number: 2019-0014).

### 4.2. Generation of Fech-Knockout Zebrafish Using CRISPR/Cas9 Technology

*fech* knockout zebrafish (*fech*^−/−^) were generated using the CRISPR/Cas9 gene-editing tool [72]. The target site for CRISPR/Cas9 was determined by Integrated DNA Technologies (IDT) (CRISPR-Cas9 guide RNA design checker | IDT (idtdna.com) (accessed on 2 April 2021). Target-specific single-guide RNA (sgRNA) was synthesized using oligos (Table 1) according to a previously described method [73]. The Cas9 protein (100 ng/µL) and sgRNA (50 ng/µL) mixture was introduced into single-cell stage embryos using a PicoPump micro-injector (World Precision Instruments, Sarasota, FL, USA). Mutagenesis efficiency was determined 24 h after micro-injection using T7 Endonuclease I (T7E1; NEB, Ipswich, MA, USA) digestion in accordance with a previously described method [74]. The primer sequences used for target site amplification are listed in Table 1. For genotyping, genomic DNA extracted from embryos, larvae, or the caudal fins of adult zebrafish was used as a template for polymerase chain reaction (PCR) heteroduplex mobility assays.

### 4.3. Assessing Phenotypic and Physiological Effects of Fech Deletion in Zebrafish Larvae

After generating the *fech*^−/−^ zebrafish larvae, experiments were conducted to investigate their morphological changes and light sensitivity to standard microscope white light, and the impact of *fech* deletion on hemoglobin production in erythrocytes was also investigated. The detailed methodologies used in this experiment are described in the Supplementary Materials).

### 4.4. Tissue Collection for Fech Tissue Distribution Analysis

To analyze the tissue distribution of *fech* mRNA, five six-month-old healthy zebrafish were anesthetized with 0.1 mg/mL (final concentration) of tricaine methane sulfonate (MS-222; Tricaine, Sigma-Aldrich, St. Louis, MO, USA), and muscle, skin, intestine, brain, heart, testes, liver, ovary, gill, kidneys, and spleen were isolated. Harvested tissues were immediately flash-frozen in liquid nitrogen and stored at −80 °C until RNA extraction. cDNA synthesis, followed by RT-qPCR, was performed to analyze the tissue distribution of *fech*.

### 4.5. Total RNA Extraction and RT-qPCR

Total RNA was isolated from pooled tissue samples or embryos using TRIzol reagent (Thermo Fisher Scientific, Waltham, MA, USA). The concentrations of extracted RNA were measured using a MultiskanTM GO Microplate Spectrophotometer (Thermo Scientific) and diluted to 500 ng/µL. The quality of RNA was determined using gel electrophoresis. A total of 3 µg of extracted total RNA was used to synthesize cDNA using a PrimeScript first-strand cDNA synthesis kit (Takara Bio Inc., Kusatsu, Japan). The synthesized cDNA was diluted 30-fold in nuclease-free water and amplified using PCR or utilized for gene expression analysis using RT-qPCR.

To perform RT-qPCR, a 10 µL reaction mixture was prepared using 3 µL of a cDNA template, 5 µL of TB Green Premix Ex Taq II, and 1 µL of forward and reverse primer mix (10 pmol/µL each). For RT-qPCR, the following conditions were employed: an initial denaturation at 95 °C for 10 s; 45 PCR cycles each consisting of denaturation at 95 °C for 5 s, annealing at 58 °C for 10 s, and extension at 72 °C for 20 s; and then one melting cycle (95 °C for 15 s, 60 °C for 30 s, and 95 °C for 15 s). Each experiment was conducted in triplicate. The transcription level was calculated by using the 2^−ΔΔCT^ method [75].

### 4.6. Whole-Mount In Situ Hybridization

WISH was performed according to a previously described method with some modifications [33]. Briefly, the zebrafish fech gene was amplified using PCR and subcloned into the pGEM-t Easy Vector (Promega, Madison, WI, USA). Sequencing was performed to confirm the sequence and antisense orientation of the cloned fech gene (Macrogen, Seoul, Republic of Korea). Using this clone as a template, a digoxigenin-labeled antisense RNA probe was synthesized via in vitro transcription. Zebrafish embryos were collected at each developmental stage according to previously described morphological criteria [76]. After 20 hpf, the collected zebrafish larvae were transferred and raised in 0.003% phenylthiourea to prevent pigmentation. The embryos collected at each life stage were fixed in 4% paraformaldehyde (PFA) in phosphate-buffered saline (PBS) overnight at 4 °C. The following day, embryos were transferred to 100% methanol and stored at 4 °C until WISH was performed. Following WISH, the embryos were stained (NBT/BCIP, Roche, Basel, Switzerland), fixed in 4% PFA in PBS, rinsed with PBS, mounted with 70% glycerin, and imaged using an Axioskop 2 Plus microscope (Zeiss, Oberkochen, Germany).

### 4.7. Visualization of PPIX Accumulation in fech^−/−^ Zebrafish Embryos and Larvae

To visualize fluorescence resulting from PPIX accumulation, WT and *fech*^−/−^ embryos and larvae raised in 0.003% phenylthiourea (PTU) solution were dechorionated and collected at 1–8 dpf. Subsequently, the collected embryos were anesthetized using 0.1 mg/mL of tricaine methane sulfonate (MS-222; Sigma-Aldrich) and examined under a red filter using a fluorescence microscope at 400× magnification (Leica DM6000 B; Leica Microsystems, Wetzlar, Germany).

### 4.8. Analyzing the Effect of UDCA on PPIX Accumulation in fech^−/−^ Larvae

To investigate the effect of UDCA (Sigma-Aldrich) on PPIX accumulation, 3 dpf WT and *fech*^−/−^ larvae raised in a 0.003% PTU solution were treated with 100 or 200 µM of UDCA dissolved in DMSO until imaging was conducted. Another set of groups was treated with DMSO alone, whereas the control groups were left untreated. Subsequently, the larvae were incubated at 28 °C, and fluorescence images were captured at 12 and 24 h after the treatments to observe PPIX accumulation and morphological changes using a fluorescence microscope at 400× magnification (Leica DM6000 B; Leica Microsystems, Wetzlar, Germany). Fluorescence intensity was calculated using ImageJ 1.54d (Wayne Rasband and contributors, National Institutes of Health, Bethesda, MD, USA).

### 4.9. Analyzing the Impact of Fech Deletion in Zebrafish on Apoptosis Using Acridine Orange Staining

The effect of fech deletion on apoptosis in zebrafish larvae at 1–7 dpf was investigated using the acridine orange staining method previously described by Tucker and Lardelli with some modifications [77].

Briefly, WT and *fech*^−/−^ embryos were raised in a 0.003% PTU solution, and embryos were transferred to the PTU solution before 20 hpf. At the specified time points, live embryos were dechorionated and subsequently incubated with a 5 µg/mL acridine orange solution in the embryo medium for 30 min. Following staining, larvae were transferred to an embryo medium and rinsed for 30 s. Subsequently, fluorescence images were visualized under a GFP filter using a fluorescence microscope at 400× magnification (Leica DM6000 B; Leica Microsystems). Relative fluorescence intensities were calculated using ImageJ software (version 1.54d, developed by Wayne Rasband and contributors, National Institutes of Health).

### 4.10. Analyzing the Effect of UDCA on Apoptosis Resulting from PPIX in fech^−/−^ Larvae Using Acridine Orange Staining

To investigate the effect of UDCA (Sigma-Aldrich) on cell apoptosis in *fech*^−/−^ larvae, 3 dpf WT and *fech*^−/−^ larvae, raised in a 0.003% PTU solution, were treated with 100 µM of UDCA dissolved in DMSO. Another set of groups was treated with DMSO alone, whereas the control groups were left untreated. Subsequently, the larvae were incubated at 28 °C, and acridine orange staining was performed 48 h after the treatment. The apoptotic cells were visualized using a fluorescence microscope at 400× magnification (Leica DM6000 B; Leica Microsystems), and relative fluorescence intensities were calculated using ImageJ software (version 1.54d, developed by Wayne Rasband and contributors, National Institutes of Health)

### 4.11. Analyzing the Impact of UDCA Treatment on the Expression of Bile Acid Transporter-Related and Apoptosis-Related Genes

To investigate the effect of UDCA on mRNA levels, 4 dpf larvae were treated with 100 µM of UDCA dissolved in DMSO, whereas another group was treated with DMSO alone the control group did not receive any treatment. Notably, 24 h after treatment, the larvae were harvested, and total RNA extraction, cDNA synthesis, and RT-qPCR were performed as previously described. The expression of bile acid transportation-related (*abcb11a* and *abcc2*) and apoptosis-related (*bax* and *bcl2*) genes was subsequently analyzed. The *bax*/*bcl2* ratio was calculated to gain further insights. The primers used in this study are listed in Table S1.

### 4.12. Analyzing the Effect of Fech Deletion on Neutrophil and Macrophage Production

To investigate whether *fech* deletion had any effect on neutrophil and macrophage production, Sudan black and neutral red stains, respectively, were applied to WT and *fech*^−/−^ embryos and larvae from 2 to 6 dpf. The total neutrophil and macrophage counts were measured in the head, trunk, and tail at each developmental stage.

Sudan black staining was performed to stain neutrophils according to the method described by Rosowski in protocol https://dx.doi.org/10.17504/protocols.io.rced2te (accessed on 7 September 2024) [78], with some modifications. Briefly, embryos or larvae were fixed in ice-cold 4% PFA in PBS for 2 h at room temperature and then rinsed in PBS. They were subsequently incubated in a Sudan black (Sigma-Aldrich) working solution in the dark for 30 min and washed extensively in 70% ethanol. Finally, the samples were rehydrated with PBS containing 0.1% Tween and imaged under an Axioskop 2 Plus microscope (Zeiss).

Neutral red staining of macrophages was conducted as described in a previous study, with some modifications [79]. Briefly, embryos and larvae were raised in 0.003% PTU and incubated in a 2.4 µg/mL (final concentration) neutral red (Sigma-Aldrich) solution in embryo medium for 4 h at 28 °C in the dark. After being stained, embryos and larvae were transferred to the embryo medium and imaged using an Axioskop 2 Plus microscope (Zeiss).

### 4.13. Analyzing the Impact of UDCA Treatment on Neutrophil and Macrophage Production in fech^−/−^ Larvae

To investigate the effect of UDCA on neutrophil production in *fech*^−/−^ larvae, 2 dpf WT and *fech*^−/−^ larvae in an embryo medium were treated with 100 µM of UDCA dissolved in DMSO. Another group of larvae in embryo medium was treated with DMSO alone, whereas the control group was left untreated. After 24 h, the larvae were stained with Sudan black, as described previously, and the total neutrophil count was determined.

Macrophage production after treating *fech*^−/−^ larvae with UDCA was examined in 4 dpf WT and *fech*^−/−^ larvae (48 h post-treatment) using the same treatments as described for neutrophil production analysis. Macrophage staining was performed as described in Section 4.12, and the total macrophage count was recorded.

### 4.14. Statistical Analysis

Each experiment was performed in triplicate. The outcomes are shown as the means ± standard deviation (SD). A one-way analysis of variance (ANOVA) was used to statistically examine the tissue distribution results. The student’s *t*-test was used to assess the statistical significance of differences between groups. Graphs were created using GraphPad Prism version 8.0.2 (GraphPad Software, Inc., San Diego, CA, USA). A *p*-value ≤ 0.05 was considered significant

In addition to the methods described above, detailed methodologies for in silico analysis, phenotypic observations of knockout larvae, assessment of the effect of *fech* deletion on hemoglobin production, and evaluation of the sensitivity of *fech*-knockout larvae to white light from conventional microscopes are included in the Supplementary Methods Section (see Supplementary Materials).

## 5. Conclusions

Utilizing *fech*^−/−^ zebrafish larvae as a model, our study provides crucial insights into erythropoiesis, PPIX accumulation, and immune responses in EPP1. We observed impaired heme biosynthesis in *fech*^−/−^ larvae, which resulted in PPIX accumulation and liver damage, thereby mirroring EPP1 characteristics. Moreover, *fech*^−/−^ larvae showed a weak effect on white light from a microscope and reduced erythrocyte production. UDCA treatment effectively reduced PPIX accumulation, apoptosis, and liver inflammation, demonstrating its therapeutic potential. These findings establish *fech*^−/−^ zebrafish larvae as a valuable model for studying EPP1 pathogenesis and drug screening. The findings of the present study have opened avenues for conducting further research on immune function and hematopoiesis in patients with EPP1.

## Figures and Tables

**Table 1:** Oligonucleotide sequences for CRISPR/Cas9-mediated *fech*-knockout.

Application		Primer Sequence (5′ to 3′)
sgRNA synthesis	T7-sgRNA (Forward)	GAAATTAATACGACTCACTATAGGAAGGAATGGTGAAACTGCgtttTagagctagaaatagcaagttAaaat
	Universal reverse primer	gatccgcaccgactcggtgccactttttcaagtTgataaCggactagccttatttTaacttgctatttctag
T7E1 assay	F	ATTGCCAAAAGACGCACCCCAAAGATC
	R	CTCACCCGTGTCCGGACACATCTCAT
Mutation confirmation (RT-qPCR)	F	GGTGAAACTGCTGGATGAGATGTGT
	R	ACTGTGGGTACTGTGTGAAGGCC

## Data Availability

The data from this study are accessible upon reasonable request from the corresponding author.

## Supplementary Materials

The following supporting information can be downloaded at https://www.mdpi.com/article/10.3390/ijms251910819/s1 [80,81,82,83,84].
