# Supplementary material for: CRISPR/Cas9-Mediated fech Knockout Zebrafish: Unraveling the Pathogenesis of Erythropoietic Protoporphyria and Facilitating Drug Screening
Source: Int J Mol Sci. 2024 Oct 8;25(19):10819. doi: 10.3390/ijms251910819 (PMC11476521; doi:10.3390/ijms251910819)
Supplement: Supplementary file 1 [file ijms-25-10819-s001.zip › ijms-3179735-supplementary.pdf]

# Supplementary materials and methods

**CRISPR/Cas9-Mediated *fech* Knockout Zebrafish: Unraveling the Pathogenesis of Erythropoietic Protoporphyria and Facilitating Drug Screening**

## 1 *In silico* analysis of Fech

The full-length of the *fech* coding sequence was extracted from the Ensemble online database (1). Protein sequences of other orthologs were obtained from the National Center for Biotechnology Information (NCBI) GenBank database (2). Using obtained sequences, multiple and pairwise sequence analysis were done using the Clustal Omega Multiple Sequence alignment tool (3) and EMBOSS Needle Pairwise sequence alignment tool, respectively (4).

## 2 Analyzing morphological and light sensitivity in *fech* knockout zebrafish larvae

We observed morphological changes in *fech*$^{-/-}$ zebrafish larvae and compared them to WT larvae using a Leica microscope (Leica DMi8 ; Leica Microsystems, Wetzlar, Germany). To investigate the light sensitivity of *fech*$^{-/-}$ larvae, we exposed WT and *fech*$^{-/-}$ larvae at 1, 3, 5, 7, and 9 dpf to the maximum light intensity (Pmax: 15W, LED light source) of the Leica microscope (Leica DM6000 B; Leica Microsystems, Wetzlar, Germany) for 20 minutes, while

another group of WT and *fech*$^{-/-}$ larvae remained unexposed. Twenty minutes after exposure, we captured images of the larvae using a Leica microscope (Leica DM6000 B; Leica Microsystems, Wetzlar, Germany).

To investigate the chronic effects of light exposure on *fech*$^{-/-}$ larvae, we subjected WT and *fech*$^{-/-}$ larvae to daily exposure of the maximum light intensity of a Leica microscope for 20 minutes. Morphological changes were then observed under the microscope after reaching 7 dpf. In addition, seven separate groups of WT and *fech*$^{-/-}$ larvae were exposed to the maximum light intensity of a Leica microscope for 20 min at 1, 2, 3, 4, 5, 6, and 7 dpf, with each group being exposed only on its respective day. Morphological changes for all these groups were observed at 7 dpf. A control group was kept unexposed, and morphological changes were observed under the Leica microscope at 7 dpf as well.

**3 Analyzing the impact of *fech* deletion on hemoglobin production in erythrocytes**

Hemoglobin staining was performed to assess the efficacy of *fech* deletion in disrupting the heme biosynthesis process in our knockout model. To achieve this, we conducted *o*-dianisidine staining following the method described by Paffet-Lugassy and Zon with some modifications (5).

In brief, the staining solution was prepared by sequentially mixing 2 mL of *o*-dianisidine solution (2,3-Diphosphoglyceric acid fast blue B) (Sigma-Aldrich), 500 µL of 0.1 M Sodium Acetate Anhydrous (NaOAc) with pH adjusted to 4.5, 2 mL of deionized $H_2O$, and 100 µL of $H_2O_2$. We then added 500 µL of the staining solution to live zebrafish embryos from 2 to 6 dpf after dechorionation, followed by incubation in the dark for 15 min.

Once the staining was completed, the stained embryos were washed with deionized $H_2O$ and fixed in 4% paraformaldehyde (PFA) for at least 1h at room temperature. After the fixation

process was completed, embryos were further washed with phosphate-buffered saline with Tween 20 (PBST) and stored at 4°C in PBST until imaging. Images were captured using an Axioskop 2 plus microscope (Zeiss, Germany).

## Supplementary results

**Table S1.** qPCR primers used in the study.

| **Gene** | | **Sequence (5'-3')** |
|---|---|---|
| ***bax*** | F | CGCCTTGTCATCAAGGCTATTTCAACC |
| | R | CCTGATCCAGTTAATGACGTGCTCC |
| ***Bcl2*** | F | GCACACTGGATGACTGACTACCTG |
| | R | CTGCTGACCGTACATCTCCACG |
| ***abcb11a*** | F | AGGGCTACTCGTTCGCTAAATCAGG |
| | R | TGGCCAATCTGGTCGTCAAAGC |
| ***abcc2*** | F | CTGGGAGAATTGCACAGCATCAAGG |
| | R | ATCGCTCCTCATCCACTGAAGAACC |
| ***ef1α*** | F | CTCCTCTTGGTCGCTTTGCT |
| | R | CCGATTTTCTTCTCAACGCTCT |
| ***fech*** | F | AAGATGGAGTGGAGAGAGCTGTGG |

| | |
|---|---|
| R | AATCACACTCCAGCGCATCTTCG |

**Table S2.** Comparing Zebrafish Fech's pairwise percentage identity and similarity to a selected vertebrate orthologs.

| Species | Accession number | Taxonomy | Identity (%) | Similarity (%) |
|---|---|---|---|---|
| ***Carassius auratus*** | XP_026052758.1 | Fish | 91.4 | 95.1 |
| ***Megalobrama_amblycephala*** | XP_048022282.1 | Fish | 91.9 | 95.6 |
| ***Colossoma_macropomum*** | XP_036443583.1 | Fish | 90.2 | 94.9 |
| ***Homo_sapiens*** | CAB65962.1 | Mammalia | 71.8 | 81.6 |
| ***Mus_musculus*** | AAH06746.1 | Mammalia | 62.8 | 72.6 |
| ***Pan_troglodytes*** | JAA36967.1 | Mammalia | 71.2 | 81.2 |
| ***Xenopus_laevis*** | NP_001081718.1 | Amphibia | 71.3 | 82.7 |
| ***Rana_temporaria*** | XP_040193453.1 | Amphibia | 70.7 | 81.4 |
| ***Chrysemys_picta_bellii*** | XP_005296886.1 | Reptilia | 73.5 | 81.3 |
| ***Chelydra_serpentina*** | KAG6926243.1 | Reptilia | 73.3 | 80.7 |
| ***Corvus_hawaiiensis*** | XP_048147475.1 | Aves | 74.0 | 82.9 |
| ***Anser_cygnoides*** | XP_013050572.1 | Aves | 73.6 | 84.1 |
| ***Tyto_alba*** | XP_042660703.1 | Aves | 72.8 | 82.3 |

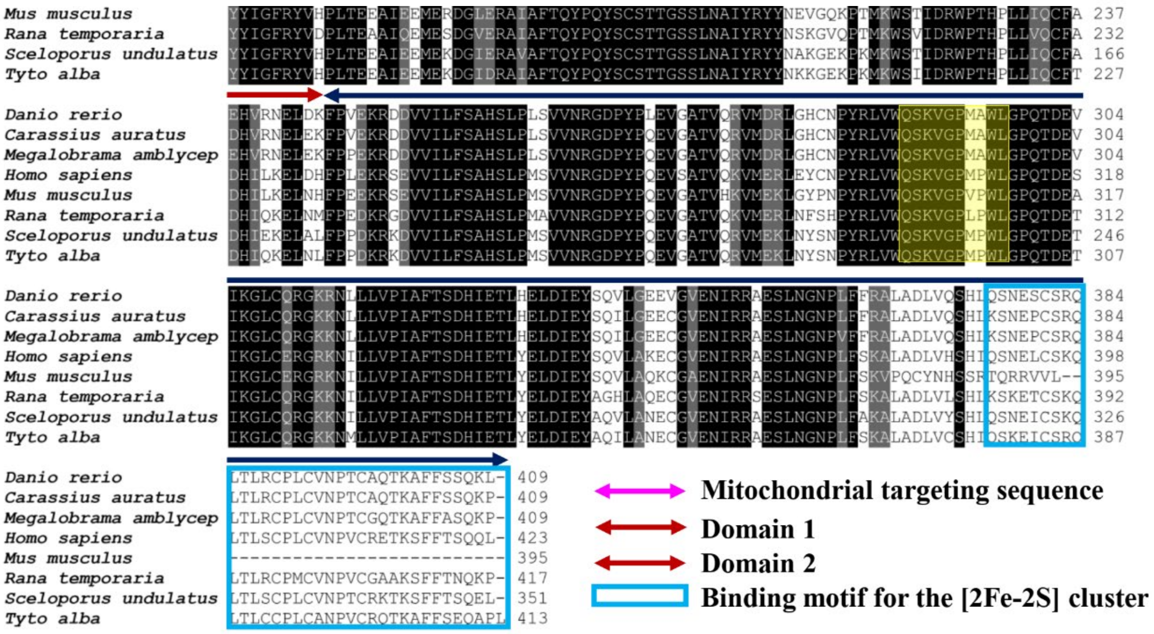

**Figure S1.** Multiple sequence alignment of Zebrafish Fech with its orthologs. The amino acids shaded in black are fully conserved, and the amino acids shaded in gray are nearly conserved. Yellow shaded regions represent the active site pockets.

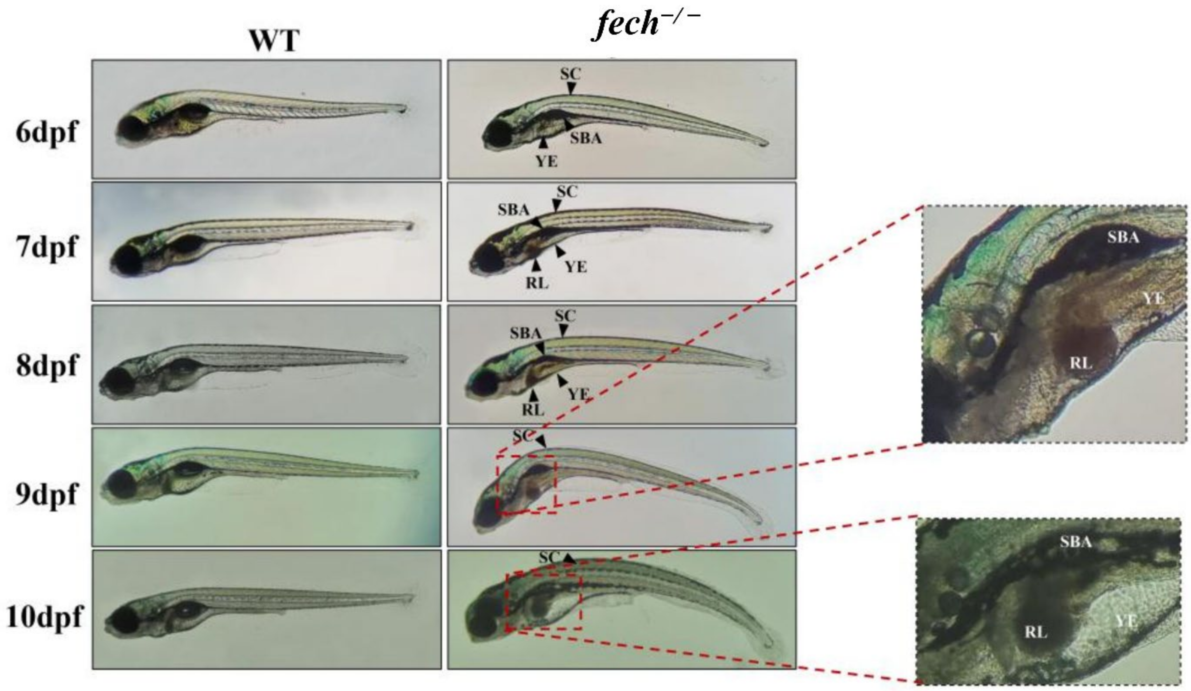


**Figure S2.** Phenotypic deformity observation of *fech*$^{-/-}$ larvae. Phenotypic analysis was identified in larvae 6 dpf onwards. (**RL**, reddish and enlarged liver; **YE**, yolk sac edema; **SC**, inward spinal curvature; **SBA**, swim bladder abnormality)

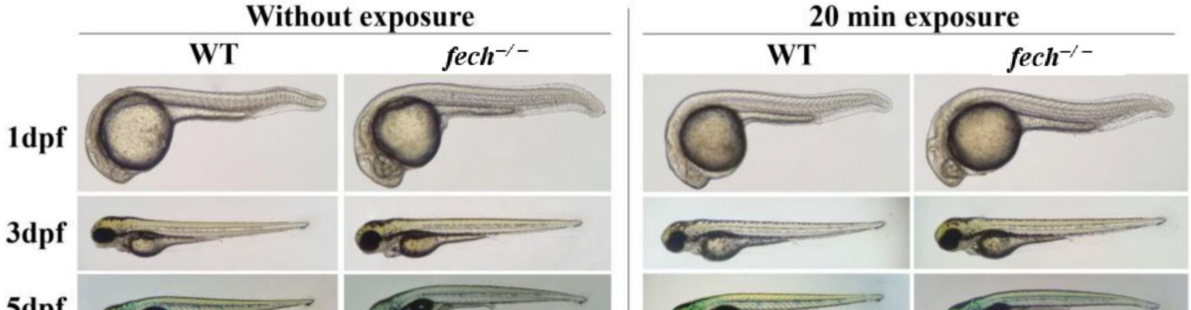

**Figure S3.** Analysis of the acute effect of standard microscopic light exposure on *fech*$^{-/-}$ larvae. 1, 3, 5, 7, and 9 dpf WT and *fech*$^{-/-}$ larvae were exposed 20 min for white light of standard microscopic light.

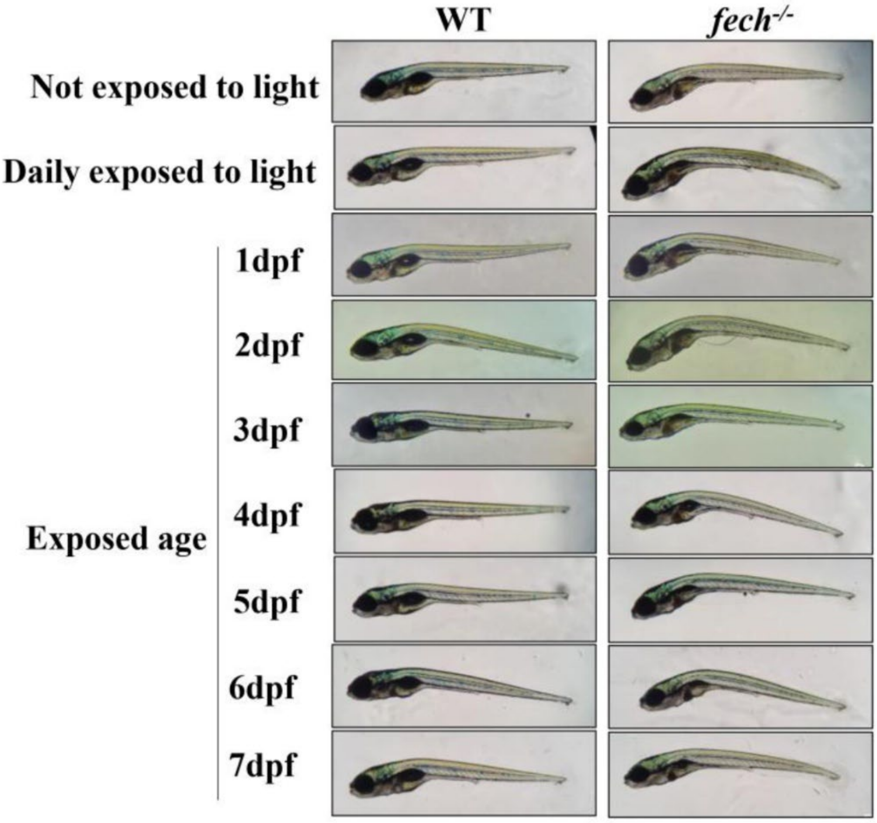


**Figure S4.** Analysis of the chronic effect of standard microscopic light exposure on *fech*$^{-/-}$ larvae

**A**

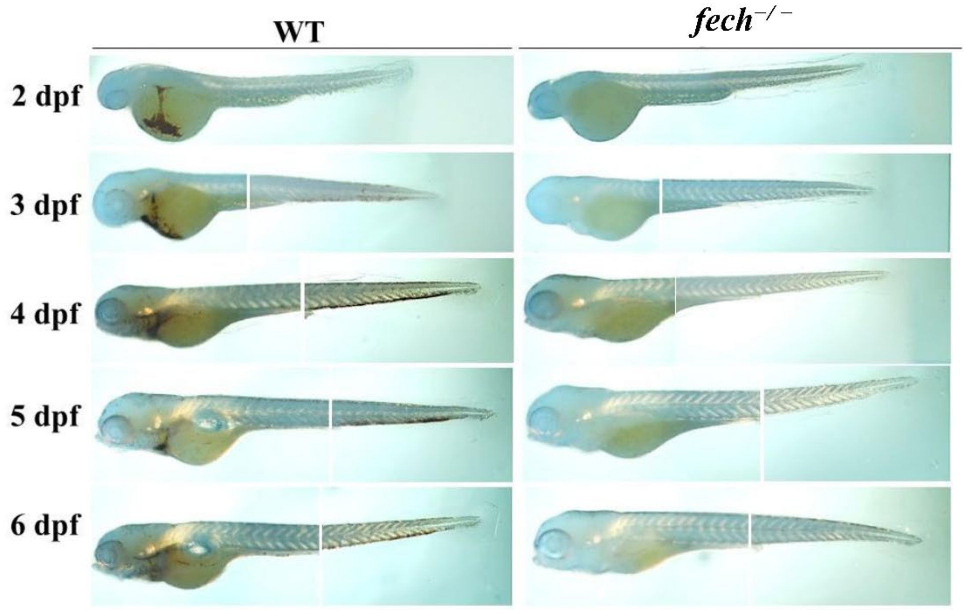
WT
*fech*−/−
2 dpf
3 dpf
4 dpf
5 dpf
6 dpf

**B**

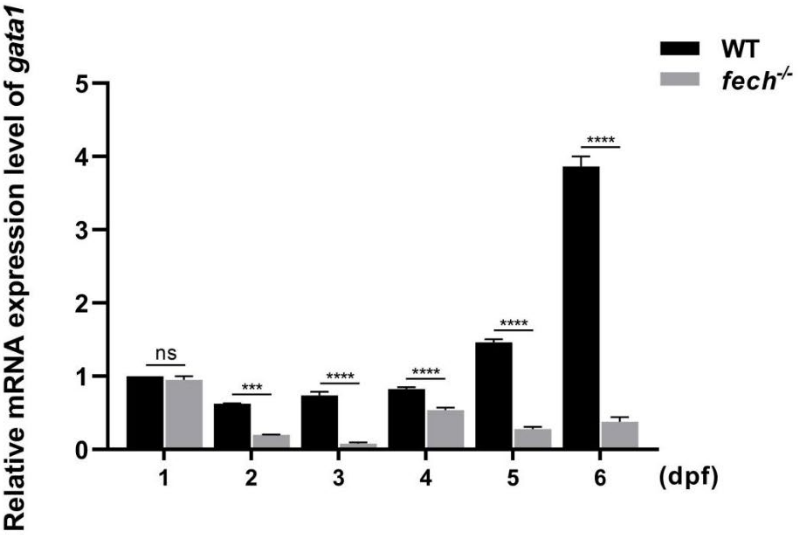
Relative mRNA expression level of *gata1*
WT
*fech*-/-
5
4
3
2
1
0
ns
***
****
****
****
****
1
2
3
4
5
6
(dpf)

**Figure S5.** Effect of *fech* deletion on heme synthesis (functional hemoglobin production) and the erythrocyte marker gene *gata1* expression in different developmental stages of *fech*$^{-/-}$ larvae. **(A)** *o*-dianisidine staining of hemoglobin in WT and *fech*$^{-/-}$ larvae. **(B)** *gata1* mRNA expression in different life stages of *fech*$^{-/-}$ zebrafish larvae. The 1 dpf larvae show no significant difference compared to the WT, but there is a reduction in mRNA expression until 3 dpf. At 4 dpf, the larvae exhibit a slight elevation of *gata1* mRNA expression, followed by a slight reduction and unchanged expression levels in 5 and 6 dpf larvae. Overall, *gata1* expression is lower in *fech*$^{-/-}$ larvae compared to the WT larvae. The RT-qPCR data are represented as mean ± SD (n=3). Statistical significance between *fech*$^{-/-}$ and WT larvae was analyzed using Student's *t*-test (ns, non-significance; $p > 0.05$; ***, $p \leq 0.001$; ****, $p \leq 0.0001$).

**References**


1. Hubbard T. The Ensembl genome database project. Nucleic Acids Research. 2002 Jan 1;30(1):38–41.

2. Benson D, Lipman DJ, Ostell J. GenBank. Nucl Acids Res. 1993;21(13):2963–5.

3. Sievers F, Higgins DG. The clustal omega multiple alignment package. In: Katoh K, editor. Multiple Sequence Alignment [Internet]. New York, NY: Springer US; 2021 [cited 2023 Oct 5]. p. 3–16. (Methods in Molecular Biology; vol. 2231). Available from: http://link.springer.com/10.1007/978-1-0716-1036-7_1

4. Rice P, Longden I, Bleasby A. EMBOSS: The european molecular biology open software suite. Trends in Genetics. 2000 Jun;16(6):276–7.

5. Paffett-Lugassy NN, Zon LI. Analysis of hematopoietic development in the zebrafish. In: developmental hematopoiesis [Internet]. New Jersey: Humana Press; 2004 [cited 2023 Oct 7]. p. 171–98. Available from: http://link.springer.com/10.1385/1-59259-826-9:171
